# A Generalized Methodology of Designing 3D SERS Probes with Superior Detection Limit and Uniformity by Maximizing Multiple Coupling Effects

**DOI:** 10.1002/advs.201900177

**Published:** 2019-04-04

**Authors:** Yi Tian, Hanfu Wang, Lanqin Yan, Xianfeng Zhang, Attia Falak, Yanjun Guo, Peipei Chen, Fengliang Dong, Lianfeng Sun, Weiguo Chu

**Affiliations:** ^1^ Nanofabrication Laboratory CAS Key Laboratory for Nanosystems and Hierachical Fabrication CAS Center for Excellence in Nanoscience National Center for Nanoscience and Technology Beijing 100190 P. R. China; ^2^ Center of Materials Science and Optoelectronics Engineering University of Chinese Academy of Sciences Beijing 100049 P. R. China

**Keywords:** localized surface plasmon resonance (LSPR), quantification of multiple coupling effects, SERS probe design, surface plasmon polariton (SPP), trace detection

## Abstract

Accurate design of high‐performance 3D surface‐enhanced Raman scattering (SERS) probes is the desired target, which is possibly implemented with a prerequisite of quantifying formidable multiple coupling effects involved. Herein, by combining theory and experiments on 3D periodic Au/SiO_2_ nanogrid models, a generalized methodology of accurately designing high performance 3D SERS probes is developed. Structural symmetry, dimensions, Au roughness, and polarization are successfully correlated quantitatively to intrinsic localized electromagnetic field (EMF) enhancements by calculating surface plasmon polariton (SPP), localized surface plasmon resonance (LSPR), optical standing wave effects, and their couplings theoretically, which is experimentally verified. The hexagonal SERS probes optimized by this methodology realize over two orders of magnitudes (405 times) improvement of detection limit for Rhodamine 6G model molecules (2.17 × 10^−11^ m) compared to the unoptimized probes with the same number density of hot spots, an enhancement factor of 3.4 × 10^8^, a uniformity of 5.52%, and are successfully applied to the detection of 5 × 10^−11^ m Hg ions in water. This unambiguously results from the Au roughness‐independent extra 144% contribution of LSPR effects excited by SPP interference waves as secondary sources, which is very unusual to be beyond the conventional recognition.

## 1 Introduction

Surface‐enhanced Raman scattering (SERS) is a very powerful technique for molecule detection which has extensive applications in chemical, biological, and environmental fields.^1^, ^2^, ^3^, ^4^, ^5^ Detection limit and uniformity are critical for SERS probes in which the detection limit is determined normally by both the intrinsic maximum localized electromagnetic field (EMF) enhancement at hot spots on plasmonic substrates, and the interaction between the probe and the molecule to be detected, being very significant especially for trace or single molecules.^6^, ^7^, ^8^ Detection uniformity relates closely to the distributions of localized EMF (hot spots) depending on the structures of SERS probes, which is rarely reported.^9^, ^10^ Normally, increasing the number of hot spots, i.e., creating interparticle nanogaps as rich as possible, is a popular and effective way to boost the total Raman intensity of a SERS probe, which however cannot usually afford to promote its detection limit.^11^, ^12^, ^13^ In contrast, reducing the gaps at hot spots and/or creating appropriate nanostructures to enhance their intrinsic localized EMF can effectively improve the detection limit but the gap reduction is greatly conditioned by the fabrication techniques available and/or the sizes of molecules to be detected.^9^, ^10^, ^14^, ^15^, ^16^ So far, no generalized methodology capable of designing 3D SERS probes for optimization and/or tailoring of detection limit and uniformity is proposed yet, which is of vital importance for trace detection and practical applications.

Localized surface plasmon resonance (LSPR) is well known to be capable of greatly enhancing the localized EMF at hot spots,^17^, ^18^, ^19^, ^20^ and surface plasmon polariton (SPP) can also afford to enhance EMF by coupling upon propagating along metal/dielectric interfaces.^21^, ^22^, ^23^ For the structure of metal nanoparticles/dielectric layer/metallic film, part of LSPR energy can be transferred to SPP while the interparticle EMF is enhanced by the energy transfer between LSPR and SPP by virtue of the underlying surface plasmon.^24^, ^25^, ^26^ For quasi‐3D plasmonic nanostructures, LSPR couplings, Fabry–Pérot (FP) resonances along the height, and Bloch wave SPP can strongly confine the electromagnetic energy, which favors the enhancement of EMF.^27^, ^28^, ^29^ Coupling effects between LSPR and SPP were also observed in specific structures, such as cavity‐based arrays,^30^ nanodish arrays,^31^ nanopillar arrays,^32^ hierarchical silver substrates,^33^ 3D multibranched nanostructures,^34^ and gold bowtie nanoantenna^35^ to enhance EMF through different mechanisms as well. However, these multiple coupling effects above are just reported qualitatively instead of quantitatively owing to the complexities.^24^, ^30^, ^32^, ^36^ Quantification of individual contributions of the coupling effects to SERS by enhancing localized EMF enables to understand the properties of SPP and LSPR better, which makes it possible to design novel high performance SERS probes.

Herein, we proposed a generalized design methodology of novel 3D SERS probes with drastically improved detection limit and uniformity by extracting the individual contributions of SPP wave interference, incident light standing wave and LSPR effects, and their couplings to intrinsic SERS effects based on 3D periodic Au/SiO_2_ hybrid models with different symmetries and geometric dimensions. By combining finite‐difference time‐domain (FDTD) calculations and mathematical analyses, geometric dimension, Au roughness, and polarization dependences of multiple effects for different nanogrids were established in which theory and SERS experiments agree very well. The hexagonal SERS probes designed using the methodology are experimentally demonstrated to achieve the optimum detection limit and uniformity.

## 2 Results and Discussion

### 2.1 Fabrication of SERS Probes and Intrinsic SERS Effects

**Figure 1:**
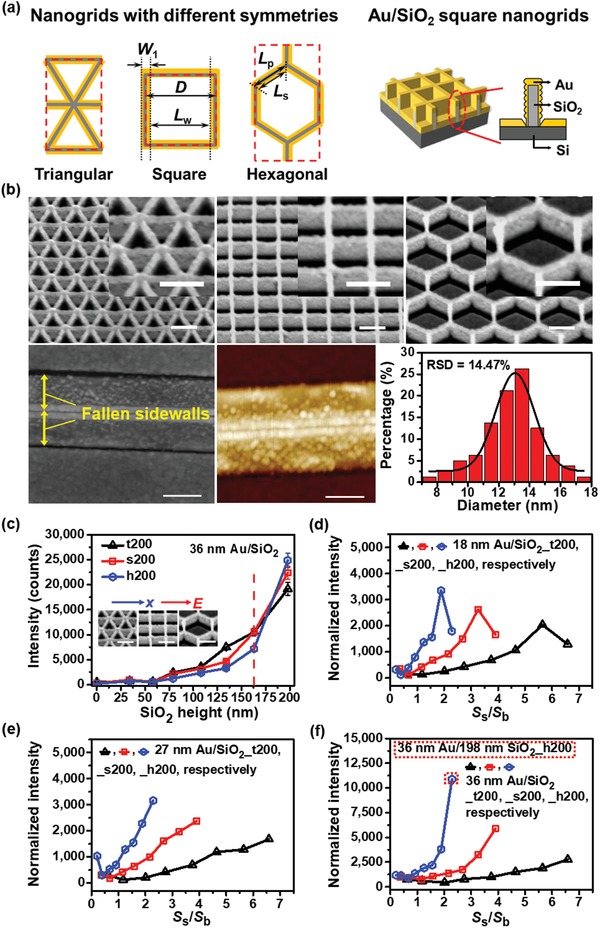
Structures of Au/SiO_2_ nanogrids and experimental SERS intensities. a) Schematics of triangular, square, and hexagonal Au/SiO_2_ periodic nanogrids. The unit cells are indicated by the red dashed rectangles. Spacing, width, and center distance of sidewalls *L*_w_, *W*_1_, and *D*, respectively, are defined in the schematics, and thus *L*_w_ = *D* − *W*_1_. Grid length *L*_p_ and sidewall length *L*_s_ are also defined in the schematics, and thus *L*_s_ ≈ *L*_p_−*W*_1_. b) Tilt SEM images of triangular, square, and hexagonal 36 nm Au/198 nm SiO_2_ nanogrids with grid length *L*_p_ of 200 nm and their corresponding enlarged images in the insets, and SEM and atomic force microscopy (AFM) (the presence of broadening effects) images of fallen 36 nm Au/198 nm SiO_2_ parallel nanowalls and their size distribution derived from these images. Scale bars: 200 nm. c) Changes of experimental SERS intensities of triangular (t200), square (s200), and hexagonal (h200) 36 nm Au/SiO_2_ nanogrids with SiO_2_ height. d–f) Normalized SERS intensities of 18, 27, and 36 nm Au/SiO_2_ nanogrids _t200, s200, and h200 by the ratio of sidewall (*S*_s_) to bottom surface area (*S*_b_) versus the ratio (*S*_s_/*S*_b_, height), respectively.

We designed and fabricated triangular (t), square (s), and hexagonal (h) 3D periodic Au decorated silicon oxide hybrid nanogrids with various grid lengths *L*_p_ and heights as SERS probe models for exploration of symmetry and geometry dependences of SERS effects.^37^, ^38^ For clarity, the sample of 36 nm thick Au deposited on hexagonal SiO_2_ nanogrids with a grid length of 200 nm and a height of 198 nm is labeled by 36 nm Au/198 nm SiO_2_ _h200. Implications of the abbreviations of samples are presented in Table S1 of Supporting Information. The defined geometric parameters of nanogrids, and their schematic fabrication and scanning electron microscopy (SEM) images are shown in **Figure** **1**a and Figures S1–S4 of Supporting Information, respectively.^39^, ^40^ Figure S4 of Supporting Information shows that the roughness of sidewalls tends to increase initially and then decrease with the increased Au thickness and/or height of sidewalls due to the lateral growth. Average 13 nm large Au nanoparticles with gaps and about average 7 nm thickness on each side of SiO_2_ nanowall (about 9 nm width), and their size distributions were statistically obtained from SEM observations on 36 nm Au/198 nm SiO_2_ nanogrids and fallen 36 nm Au/parallel SiO_2_ nanowalls, as revealed by Figure 1b. Height dependences of sidewall width and cross sectional Au area were experimentally established to obtain the density of Au nanoparticles for FDTD calculations (Section S1, Figure S5, and Table S2, Supporting Information). The larger the thickness of Au is, the more rapidly the Au area increases with height, implying the more effective deposition of Au on the more rough sidewalls.

To investigate SERS effects, Rhodamine 6G (R6G) molecules are employed for the purpose of assessing the intrinsic performance of different SERS probes.^14^, ^15^ Height dependences of intensity of the peak at 1360 cm^−1^ of R6G for Au/SiO_2_ nanogrids _t200, s200, and h200 with different Au thicknesses for the *x*‐polarized light are presented in Figure 1c and Figure S6 of Supporting Information. For 36 nm Au thickness, the intensity increases more rapidly as the height of SiO_2_ nanogrids increases, which probably results from the increase of sidewall roughness and/or extra effects with height. The intensities of the reference samples without sidewalls (i.e., the height is zero) are low enough to be negligible. However, for the same height with different thicknesses of 9, 18, and 27 nm (Figure S6, Supporting Information) the intensity decreases as t200 > s200 > h200, whereas for 36 nm the intensity decreases still as t200 > s200 > h200 for the heights lower than 163 nm and as h200 > s200 > t200 for the height of 198 nm. Normally, the SERS intensity is proportional to the number density of hot spots (the number of hot spots per unit area), which is evidenced by the change of t200 > s200 > h200 for the heights lower than 163 nm. However, the anomalous intensity change here for the heights larger than 163 nm leads one to infer reasonably that different SERS effects for different nanogrids arise predominantly from different geometries. Thus, intrinsic SERS effects for different geometries can be evaluated using the SERS intensities normalized by their corresponding ratios of sidewall to bottom area, *S*_s_/*S*_b_, which is basically proportional to the number density of hot spots, as shown in Figure 1d–f. Interestingly, the normalized intensity for h200 (the lowest number density of hot spots) with 36 nm thick Au increases most rapidly with *S*_s_/*S*_b_ whereas that for t200 (the highest number density of hot spots) increases most slowly. This reveals that the strongest intrinsic SERS effect for h200 especially for the larger heights cannot be attributed to the rough sidewalls simply but extra effects produced by the hexagonal geometry.

### 2.2 Multiple Coupling Effects Involved

**Figure 2:**
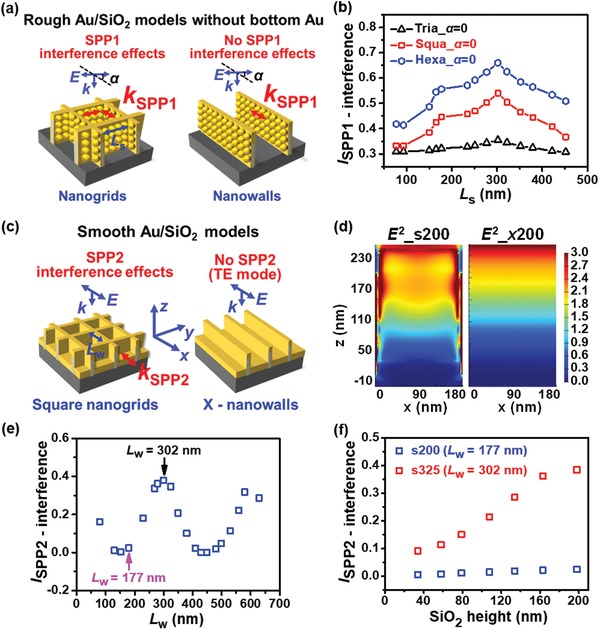
Geometric dimension dependences of SPP effects derived from FDTD calculations based on different 3D Au/SiO_2_ models. a) 3D models of the rough square Au/SiO_2_ nanogrids and nanowalls without bottom Au. The comparison of the effects calculated from two models allows one to derive the SPP1 wave interference effect. b) Calculated interference intensities of SPP1 wave excited at the rough sidewalls of triangular, square, and hexagonal 36 nm Au/198 nm SiO_2_ nanogrids with the polarization angles of *α =* 0 against the increased sidewall length *L*_s_. c) 3D models of the smooth square Au/SiO_2_ nanogrids and *x*‐nanowalls. The comparison of the effects produced by two models allows for the derivation of the SPP2 wave interference effect. d) Calculated spatial distributions of the squared electric field intensity at surfaces (parallel to the *xz* plane) of the smooth square 36 nm Au/198 nm SiO_2_ nanogrids _s200 and *x‐*nanowalls *x*200 for the *x*‐polarized light. e) Calculated interference intensities of SPP2 wave for the smooth square 36 nm Au/198 nm SiO_2_ nanogrids with *L*_w_. f) Changes of calculated SPP2 interference intensity for the square nanogrids _s200 (*L*_w_ = 177 nm in which *L*_w_ = *D* − *W*_1_ with *W*_1_ = 9 nm + 7 × 2 nm = 23 nm) and s325 (*L*_w_ = 302 nm), with SiO_2_ height for the *x*‐polarized light.

**Figure 3:**
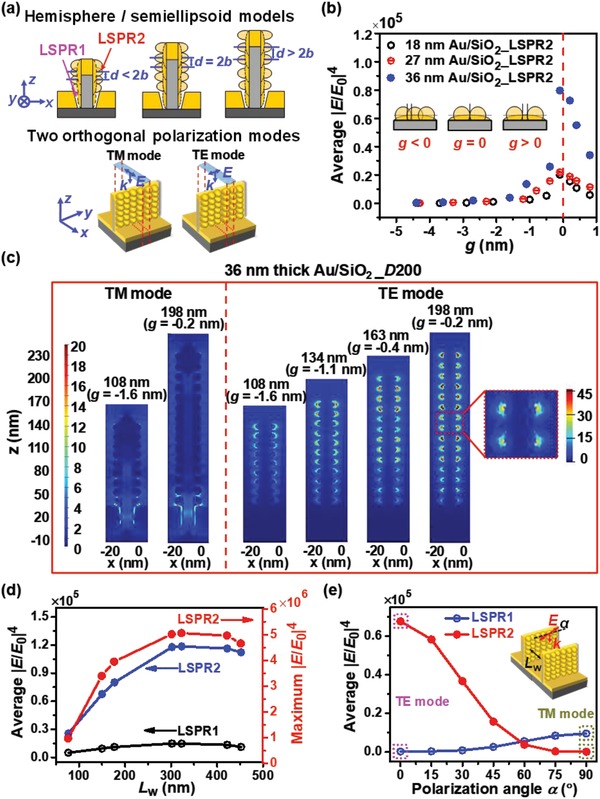
Calculated geometric dimension and polarization dependences of LSPR effects. a) The models of hemisphere/semiellipsoid Au nanoparticles for the rough *y*‐nanowalls with the increased height, accompanied by the initial increase and subsequent decrease in roughness, and the 3D rough *y‐*nanowall models with TM and TE mode for the *x* and *y* polarization, respectively, for FDTD calculations. The unit cells are indicated by the red dashed lines. b) Gap *g* dependences of the averaged fourth power of local electric field intensities of the rough nanowalls with *D* = 200 nm for TE mode for 18, 27, and 36 nm thicknesses. c) Calculated spatial distributions of the electric field intensities at the cross sections parallel to the *xz* plane of the rough *y*‐nanowalls with 200 nm center distance *D* and 36 nm thickness for different heights with TM and TE modes. Semiellipsoid Au nanoparticles have the average semiprincipal axes, *a = b =* 6.5 nm, and *c* = 8.0 nm. The hot spots at sidewall corresponding to the maximum intrinsic EF enhancement indicated by the red dashed rectangle are shown in the right panel. d) Sidewall spacing *L*_w_ dependences of the averaged |*E*/*E*_0_|^4^ of LSPR1 and LSPR2 with TM and TE mode, respectively, for the rough *y*‐nanowalls with 36 nm Au/198 nm SiO_2_, and the maximum |*E*/*E*_0_|^4^ of LSPR2 for these nanowalls for TE mode (right *y*‐coordinate). e) Calculated average |*E*/*E*_0_|^4^ of LSPR1 and LSPR2 versus polarization angle α for the rough 36 nm Au/198 nm SiO_2_ nanowalls with *L*_w_ = 151 nm. The inset shows the model for FDTD calculations. TE and TM modes correspond to 0° and 90°, respectively.

For rough Au on periodic SiO_2_ nanogrids, SPP waves and their interference effects, LSPR effects, standing wave effects of incident light, and coupling effects between LSPR and SPP are probably involved, which all may contribute to intrinsic SERS effects. SPP1 can be excited at both the Au/air and Au/SiO_2_ interfaces of rough sidewalls by a polarized light with both TE and TM modes,^41^ and SPP2 at the bottom Au/air interface with TM mode due to the periodic sidewalls,^41^, ^42^ as schematically illustrated in **Figures** **2**a,c and Figure S7a of Supporting Information. LSPR1 and LSPR2 can be excited at the gaps between bottom and sidewalls, and at those on rough sidewalls, respectively, as shown in **Figure** **3**a. The bottom Au film can reflect the incident light to form the optical standing wave, which may lead to the fluctuating distribution of electric field (EF) along the vertical height direction.^43^ SPP1 wave at the sidewalls (SPP2 wave at the bottom) can interfere with itself to form the interference wave along the horizontal direction due to the periodic cavity structures, which can further excite extra LSPR as secondary sources. Here, the incident light is taken as primary source, and then the total intensity is the sum of those excited by the primary and secondary sources.

#### 2.2.1 Dimension and Polarization Dependences of SPP Wave Interference Effects

SPP could interfere in a cavity due to multiple reflections, like FP resonance.^44^, ^45^ Their wavelengths at the Au/air and Au/SiO_2_ interfaces are derived to be 603 and 389 nm under the excitation of a 632.8 nm laser, respectively (see Section S2 and Figure S7, Supporting Information). The combination of a bottom Au‐free rough nanogrid model with SPP1 interference effects and a nanowall model without SPP1 interference effects in Figure 2a enables to derive SPP1 wave interference effects for TE mode (α = 0° and α = π/2 for TE and TM mode, respectively, with α defined as the angle between the nanowalls and the polarization direction of light) by differentiating their FDTD calculation results, which are shown in Figure 2b. The ratios of the averaged fourth power of EF enhancements^6^, ^7^ (proportional to SERS intensities, Figure S8, Supporting Information) at one sidewall of the rough triangular, square, and hexagonal 36 nm Au/198 nm SiO_2_ nanogrids to those of nanowalls, i.e., (1 + *I*_SPP1–interference_)/(1 + *I*_SPP1_) against sidewall lengths *L*_s_, can be derived for α = 0 and π/2, as shown in Figure S9a of Supporting Information. Here, the intensity of the primary incident light is set to be 1 for all calculations, and *I*_SPP1_ and *I*_SPP1–interference_ are SPP1 wave and its interference intensities, respectively. Combining the relationship of *I*_SPP1–interference_/*I*_SPP1_ versus *L*_s_ in Figure S7b of Supporting Information, we derived the sidewall length *L*_s_ dependences of *I*_SPP1–interference_ for α = 0 and π/2 to be shown in Figure 2b and Figure S9b, Supporting Information, respectively. Clearly, *I*_SPP1–interference_ for triangular, square, and hexagonal nanogrids changes against sidewall length *L*_s_ with the maxima all achieved at *L*_s_ = 302 nm. This is actually the total resultant interference effects of SPP1 waves at the Au/air and Au/SiO_2_ interfaces with the maxima achieved at *L*_s_ = 302 and 195 nm (the integral multiple of corresponding half λ_SPP1_, Figure S7b, Supporting Information), respectively, similar to the geometrical conditions that the FP resonance occurs.^29^, ^44^, ^45^ In addition, it can be seen that *I*_SPP1–interference_ also changes with α for the model of one sidewall for triangular, square, and hexagonal 36 nm Au/198 nm SiO_2_ nanogrids with *L*_s_ = 302 nm, which can be well fitted by using the expression of (*I*_1,π/2_ − *I*_1,0_)sin^2^α + *I*_1,0_ (*I*_1,π/2_ and *I*_1,0_ are the interference intensities of SPP1 wave for α = π/2 and 0, respectively) (Figure S9c, Supporting Information). Thus, the quantitative relationships between SPP1 interference effects and sidewall length *L*_s_ are well established for one sidewall of triangular, square, and hexagonal nanogrids.

Similarly, SPP2 wave interference effects can also be extracted by differentiating the FDTD calculation results on the smooth periodic nanogrid model with SPP2 effects and the *x*‐nanowall model without SPP2 effects in Figure 2c. The distributions of the squared EF intensities (i.e., light intensities) at sidewalls (*xz* plane) for the periodic square nanogrid model (*E* ^2^_s200) and for the *x‐*nanowall model (*E* ^2^_*x*200) with *D* = 200 nm are shown in Figure 2d. The ratio of *E* ^2^_s200 to *E* ^2^_*x*200 equals (1 + *I*_SPP2–interference_)/1 from which *I*_SPP2–interference_ is derived to be shown in Figure 2e. Clearly, we successfully obtain the quantitative relationships between SPP2 interference effects and sidewall spacing *L*_w_ in which *I*_SPP2–interference_ oscillates with *L*_w_, as done by the FP resonance.^44^, ^45^

Then, the vertical height dependences of the electric field intensity are mathematically derived (Section S3 and Figure S10, Supporting Information) to show the maximum normalized intensity achieved at the height of 228 nm (36% of the incident light wavelength of 632.8 nm), close to the maximum height of the fabricated Au/SiO_2_ nanogrids, 198 nm. The fluctuation in intensity along the height in Figure S10 of Supporting Informationis caused by the optical standing wave effect.^43^ This reveals for the first time that the height of a SERS probe structure, i.e., 36% of the incident light wavelength employed, is adequate for the achievement of the maximum normalized EF intensity, and further increase in height would lead to a gradually decaying oscillation of normalized EF intensity. Based on the changes of the average squared EF intensities of s200 and *x*200, and s325 and *x*325 against SiO_2_ height (Figure S11, Supporting Information), the quantitative height dependences of SPP2 interference intensity are derived to be shown in Figure 2f. The weak (strong) height dependence of SPP2 interference intensity for s200 (s325) is attributed to the destructive (constructive) interference of SPP2 occurring at *L*_w_ = 177 nm (*L*_w_ = 302 nm) for s200 (s325), as Figure 2e displays. Therefore, the SPP2 interference effects are dependent on not only the horizontal dimension but also the vertical height, especially in the case of large heights.

#### 2.2.2 Roughness, Dimension, and Polarization Dependences of LSPR Effects

SERS intensity is normally recognized to be greatly influenced by the EMF enhancement from LSPR which is dependent on the size, gap, and morphology of neighboring particles.^17^, ^18^, ^19^ Here, we reasonably adopt the relationships between center spacing *d* and radius (hemispheres for 18 nm thickness)/semi‐major (semiellipsoids for 27 and 36 nm thicknesses) *b*, i.e., *d* < 2*b* (intersected), *d* = 2*b* (tangent) and *d* > 2*b* (separated) based on SEM observations to describe the relationships between neighboring Au nanoparticles and further the change of sidewall roughness with height,^46^ as shown in the upper panel of Figure 3a. The Au particle number *n*, *d*, and the gap *g* (*g* = *d*−2*b*) between hemispheres/semiellipsoids are derived from statistical analysis and calculations of particles based on both SEM and AFM observations (Figure 1b, Figure S5 and Table S3, Supporting Information). Figure 3b shows gap *g* dependences of the averaged EF enhancements |*E*/*E*_0_|^4^ by LSPR2 for 18, 27, and 36 nm Au/SiO_2_ nanowalls _*D*200 (*D* = 200 nm) with TE mode in the lower panel of Figure 3a regardless of the coupling effects of the SPP1 waves and the optical standing wave with LSPR. The averaged |*E*/*E*_0_|^4^ reaches the maximum for *g* → 0 and drops rapidly with *g* away from zero (the smaller the absolute gap is, the larger the roughness of Au is for the same thickness). We should point out here that quantum correction does not need to be taken into account upon calculations for the infinite small gap because the Au nanoparticles with different neighboring relationships always reside on a continuous Au film to allow for the neglection of the tunneling effect of electrons occurring possibly among neighboring nanoparticles.^47^, ^48^ For the same *g* the averaged |*E*/*E*_0_|^4^ increases more rapidly for the larger thickness because of the larger roughness arising from the lateral growth of more Au. Definitely, the particle models with different gaps proposed above can unambiguously describe the change of sidewall roughness with Au thickness despite the normally inevitable size distributions of Au nanoparticles in real cases. Our purpose is to describe the change of sidewall roughness correctly by using the different models of Au nanoparticles with the average size instead of the distributed sizes.

The parameters of Au nanoparticles obtained above allow to derive height dependences of LSPR effects for 36 nm Au/SiO_2_ parallel rough nanowalls _*D*200 for TM and TE mode in the lower panel of Figure 3a by FDTD calculations, which are shown in Figure 3c. In the case of the couplings of SPP1 and SPP2 with LSPR1 for TM mode, LSPR effects are almost independent of height with the maximum localized EF enhancement of ≈20 whereas in the case of the coupling of SPP1 with LSPR2 for TE mode, LSPR effects are definitely height dependent with the maximum EF enhancement of ≈46 (the enlarged image shown in the right panel of Figure 3c) achieved at the height of 198 nm. Close inspection of Figure 3c allows one to find that the maximum intrinsic EF enhancement occurs at the hot spots positioning at around 150 nm corresponding to the antinode of the optical standing wave with the maximum amplitude.^43^ Also, the EF enhancement distributions for 18 and 27 nm thicknesses with various heights are shown in Figure S12 of Supporting Information. The above calculations reveal the optimum combination of Au thickness and SiO_2_ height, i.e., 36 nm Au thickness and 198 nm SiO_2_ height, to achieve significant EMF enhancement in the present study, which is also supported strongly by the experiments in Figure 1c–f. The difference of the EF enhancement at the gaps of Au nanoparticles for different heights in Figure 3c and Figure S12 of Supporting Information arises mainly from the optical standing wave effects (Figure S10 and Figure S11, Supporting Information).

Based on the above calculations sidewall spacing *L*_w_ dependences of the averaged |*E*/*E*_0_|^4^ from LSPR1 and LSPR2 at TM and TE mode for 36 nm Au/198 nm SiO_2_ rough nanowalls are given in Figure 3d. The average |*E*/*E*_0_|^4^ from both LSPR1 and LSPR2 achieves the maxima at *L*_w_ ≈300 nm with far stronger LSPR2, and the maximum |*E*/*E*_0_|^4^ from LSPR2, i.e., the strongest intrinsic localized EF also occurs at *L*_w_ ≈300 nm for the rough nanowalls with TE mode. To minimize the SPP2 interference effects (Figure 2e), *L*_w_ = 151 nm is taken to establish the polarization dependences of EF enhancement for 36 nm Au/198 nm SiO_2_ rough nanowalls, as shown in Figure 3e. The EF enhancements from LSPR1 and LSPR2 increase and decrease with α, respectively, and their normalized |*E*/*E*_0_|^4^ against α can be well fitted with sin^4^α and cos^4^α, respectively (Figure S13, Supporting Information). Therefore, the LSPR effects are influenced significantly by the polarization angle of α for the rough nanowall model. However, this implies that the influences would be probably smeared out for the structures with a high symmetry.

### 2.3 Effects of Nanogrid Symmetry, SPP Wave Coupling Excitation Contributions, and SERS Probes Optimizing

**Figure 4:**
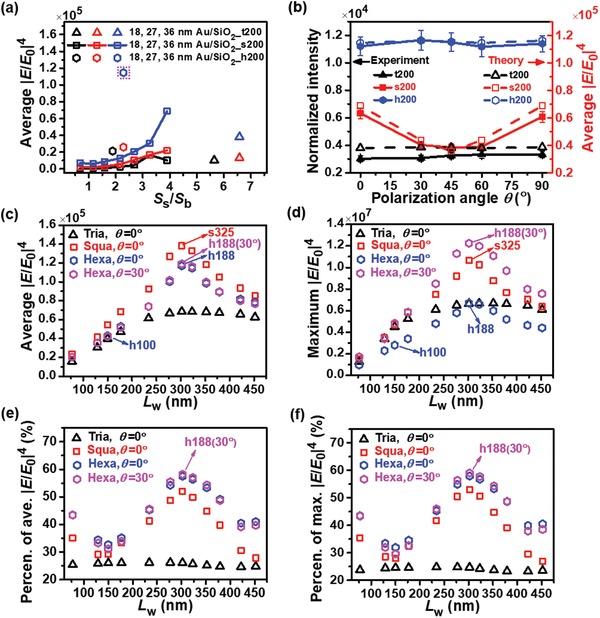
Height dependences of the total average |*E*/*E*_0_|^4^ for different nanogrids with multiple coupling effects considered, experimental and calculated polarization dependences of SERS effects for different nanogrids with different symmetries, and calculated sidewall spacing *L*_w_ dependences of total average and maximum |*E*/*E*_0_|^4^ and contributions of SPP coupling excitation effects. a) Calculated total average |*E*/*E*_0_|^4^ versus height (*S*_s_/*S*_b_) for triangular, square, and hexagonal nanogrids with 200 nm grid length, 18 nm Au/163 nm SiO_2_, 27, and 36 nm Au/198 nm SiO_2_, respectively and square 18, 27, and 36 nm Au/SiO_2_ nanogrids _s200 for the *x*‐polarized light with multiple coupling effects considered. b) Comparison of polarization angle θ dependences of the normalized experimental Raman intensities and the calculated average |*E*/*E*_0_|^4^ for 36 nm Au/198 nm SiO_2_ nanogrids _t200, s200, and h200. Experiments and calculations agree very well. The polarization angle θ is defined in the inset. Scale bars: 200 nm. c,d) Sidewall spacing *L*_w_ dependences of the calculated average and maximum |*E*/*E*_0_|^4^, respectively for triangular, square and hexagonal 36 nm Au/198 nm SiO_2_ nanogrids with multiple coupling effects considered. e,f) Sidewall spacing dependences of the total contributions of SPP wave coupling excitation effects to the average and maximum |*E*/*E*_0_|^4^, respectively.

We have successfully established the quantitative dependences of the SPP interference effects, LSPR effects, SPP and LSPR coupling effects, and the standing wave effects on the geometric dimensions and on the polarization direction based on the square nanogrid and parallel nanowall models. To generalize and extend the above analysis method to the triangular and hexagonal nanogrids, we reasonably take a triangular (square, hexagonal) nanogrid consisting of three (two couples, three couples) nanowalls with 60° (90°, 120°) relative to each other, and decompose a linearly polarized incident light into two orthogonal components parallel and normal to each nanowall, i.e., TE and TM mode, respectively, which are schematically illustrated in Figure S14 of Supporting Information. Thus, the total average |*E*/*E*_0_|^4^ for triangular, square, and hexagonal nanogrids can be derived by summing up the average |*E*/*E*_0_|^4^ from LSPR1 (TM mode) and LSPR2 (TE mode) (Figure 3d) multiplied by their respective coupling coefficients associated with the dimension and symmetry (Section S4, Table S4 and Figure S15, Supporting Information). The height (*S*_s_/*S*_b_) dependences of the total average |*E*/*E*_0_|^4^ for 18, 27, and 36 nm Au/SiO_2_ nanogrids _t200, s200, and h200 are shown in **Figure** **4**a. The calculated results are found to agree very well with the SERS experiments given in Figure 1d–f, which reveals the correctness of the models and the validity of decomposing the polarization direction of incident light into the orthogonal components parallel and normal to each sidewall for the different nanogrids.

It has been demonstrated above that the polarization of incident light has a definite effect on both LSPR and SPP effects and thus on SERS effects. Therefore, the polarization dependence of structure‐associated SERS effects would also exert an influence on the detection uniformity of a probe which is very important for molecular detection, especially trace detection. Polarization (polarization angle, θ is defined as the angle between the polarization of incident light and *x*‐direction) dependences of intrinsic SERS effects for t200, s200, and h200, along with their corresponding experimental ones, are shown in Figure 4b for comparison. Theoretical results describe the experiments very well. For both t200 and h200 nanogrids very weak θ dependences are observed, which relates closely to the sixfold symmetry involved in their structures. However, the symmetry with respect to θ = 45° is seen for s200 with the maxima at both *θ =* 0° and 90°, and the minimum at *θ =* 45°, which is easily understood in terms of its fourfold symmetry. The structures with a sixfold symmetry show the far weaker polarization dependences of structural coefficient compared to those with a fourfold symmetry. The polarization dependences of intrinsic SERS effects are determined by the polarization dependences of structural coefficients which are closely coupled to the symmetry of structures (Table S4 and Figure S15, Supporting Information). Therefore, the structures with a higher symmetry show a weaker polarization dependence of intrinsic SERS effects, which would undoubtedly promote the detection uniformity.

So far, very good agreement between theory and experiment for both height and polarization dependences of intrinsic SERS effects verifies the methodology proposed in this study. The intrinsic average and maximum |*E*/*E*_0_|^4^ determine the normalized SERS intensity. For the detection of sufficiently high concentration of molecules, the average |*E*/*E*_0_|^4^ normally plays a key role because the majority of hot spots decorated with molecules contribute to the SERS intensity. However, for the extremely low concentration of molecules (trace molecules) the hot spots with the maximum |*E*/*E*_0_|^4^ which determines the detection limit would play a far more dominant role. Therefore, for each sidewall spacing *L*_w_ there are an average intrinsic |*E*/*E*_0_|^4^ and a maximum intrinsic |*E*/*E*_0_|^4^ available in which the former may be more crucial for detection of molecules with high concentrations and the latter is more important for detection of trace molecules with extreme low concentrations. Thus, we calculated sidewall spacing *L*_w_ dependences of both the average and maximum |*E*/*E*_0_|^4^ for triangular, square, and hexagonal 36 nm Au/198 nm SiO_2_ nanogrids with all multiple coupling effects considered, which are presented in Figure 4c,d. For all the nanogrids both the average and maximum |*E*/*E*_0_|^4^ achieve the maxima at *L*_w_ = 302 nm, i.e., λ_SPP Au/air_/2 in which the summit of the average |*E*/*E*_0_|^4^ for the square nanogrids are the highest. In contrast, the summit of the maximum |*E*/*E*_0_|^4^ for the hexagonal nanogrids with a polarization angle of 30° is the highest owing to its maximum SPP coupling effects. Thus, the hexagonal 36 nm Au/198 nm SiO_2_ nanogrids _h188 with the polarization angle of 30° should have the best detection limit theoretically. The total contributions from SPP1 and SPP2 coupling effects are given in Figure 4e,f, respectively, and their respective contributions are shown in Figure S16 of Supporting Information. The maximum contributions of SPP coupling effects for the hexagonal nanogrids at *L*_w_ = λ_SPP Au/air_/2 with the polarization angle of 30° are 58.2% and 59.0% for the average and maximum |*E*/*E*_0_|^4^, i.e., 139% and 144% those without these effects, respectively, independent of Au roughness but related to plasmonic metals, beyond the conventional recognization. The optimization of structure and dimension can lead to at least one order of magnitude increase in the maximum |*E*/*E*_0_|^4^, as shown in Figure 4d, which is expected to improve the detection limit significantly. The quantitative analysis of multiple coupling effects in 3D Au/SiO_2_ periodic nanogrids is given in Table S5 of Supporting Information.

### 2.4 Detection of Trace R6G Molecules and Hg Ions

**Figure 5:**
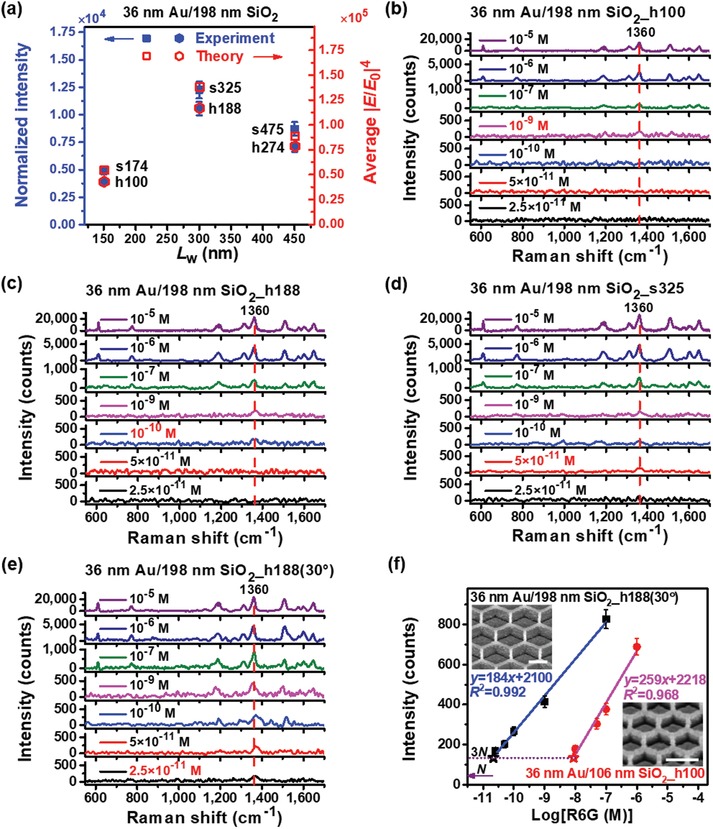
Applications of designed SERS probes for detection of trace R6G molecules. a) Comparison of the normalized Raman intensities of the peak at 1360 cm^−1^ for R6G molecules with concentration of 10^−5^ m and calculated average |*E*/*E*_0_|^4^ for square nanogrids _s174, s325, and s475, and hexagonal nanogrids _h100, h188, and h274. b–e) Raman spectra of R6G molecules with concentrations ranging from 2.5 × 10^−11^ to 10^−5^ m decorated on 36 nm Au/198 nm SiO_2_ nanogrids of h100, h188, s325, and h188 (θ = 30°), respectively. The detectable concentrations of these SERS probes are about 10^−9^, 10^−10^, 5 × 10^−11^, and 2.5 × 10^−11^ m, respectively. f) The calibration curves of 36 nm Au/106 nm SiO_2_ nanogrids of h100 and 36 nm Au/198 nm SiO_2_ nanogrids of h188 (30°) with the same number density of hot spots, i.e., the intensity of the peak at 1360 cm^−1^ versus the logarithmic concentration of R6G molecules, and their SEM images. Scale bars: 200 nm.

Thus far, quantitative dependences of multiple LSPR and SPP effects on geometric dimension, symmetry, polarization, and roughness are presented from which a generalized methodology can be developed for accurate design of the dimensions of SERS probe structures with maximum coupling effects. SEM images of s325 and h188 (36 nm Au/198 nm SiO_2_ square and hexagonal nanogrids, respectively) SERS probes designed and optimized using the methodology are shown in Figure S17 of Supporting Information to be compared with those of s174, s475, h100, and h274. Their theoretical average |*E*/*E*_0_|^4^ and normalized experimental SERS intensities agree quite well, as shown in **Figure** **5**a. The SERS mapping for the optimized h188 with a polarization angle of 30° (h188 (30°)) gives a low relative standard deviation (RSD) of 5.52% due to its high symmetry (Figure S18, Supporting Information), normally much smaller than those of the reported SERS substrates,^13^, ^49^, ^50^ which is very significant for SERS probes. h188 (30°) is revealed to show the maximum |*E*/*E*_0_|^4^ in Figure 4d and thus has the best detection limit. Experimentally, the detectable concentration of the SERS probes for R6G decreases as h100 > h188 > s325 > h188 (30°) for 36 nm Au/198 nm SiO_2_ (Figure 5b–e), which agrees very well with the change of the maximum |*E*/*E*_0_|^4^ in Figure 4d. Though s325 has the highest average |*E*/*E*_0_|^4^ in Figure 4c and can detect a concentration as low as 5 × 10^−11^ m R6G, its detection limit is not the best, further demonstrating that the detection limit is determined by the maximum |*E*/*E*_0_|^4^ (Figure 4d) instead of the average |*E*/*E*_0_|^4^, i.e., the maximum intrinsic EMF enhancement. Therefore, we can design different SERS probes upon different performance requirements by different applications. To assess the detection limit, the calibration curves of the optimized sample of 36 nm Au/198 nm SiO_2_ _h188 (30°) and the sample of 36 nm Au/106 nm SiO_2_ _h100 with the same number density of hot spots are given in Figure 5f from which their detection limits were derived to be 2.17 × 10^−11^ m and 8.78 × 10^−9^ m, respectively, according to the definition of the detection limit, i.e., the concentration with the signal‐to‐noise ratio equal to 3.^51^, ^52^ Therefore, the detection limit of 36 nm Au/198 nm SiO_2_ _h188 (30°) has an improvement of 405 times compared to that of 36 nm Au/106 nm SiO_2_ _h100 with the same number density of hot spots. Furthermore, h188 (30°) has a SERS enhancement factor (SERS EF) of 3.4 × 10^8^ (Section S5, Supporting Information), which proves theory well, being outstanding among those reported.^13^, ^30^, ^50^, ^53^

**Figure 6:**
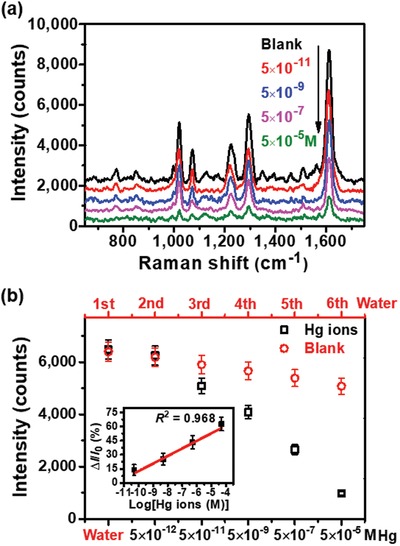
Applications of trace Hg ions detection by h188 (30°) probes. a) Raman spectra of Bpy molecules with different Hg ions concentrations. b) The relationships between Raman signal intensities (at 1610 cm^−1^) and concentrations of Hg ions and the blank control group of deionized water.

Herein, we also applied the h188 (30°) probe to detection of heavy metal Hg ions which coordinate with 4,4′‐Bipyridine (Bpy) far more strongly than Au@Ag nanoparticles and Bpy do.^54^ 5.0 × 10^−11^ m (10 ppt, about 10^2^ lower than 10^‐8^ m or 2000 ppt for US standard value for drinkable water) Hg ions was successfully detected with a good linear relationship (*R* ^2^ = 0.968) based on the drop of Raman signals of Bpy, which is shown in **Figure** **6** and Experimental Section. Therefore, the designed SERS probes using the methodology proposed were experimentally demonstrated to have the superior performance for practical detections.

So far we successfully develop a generalized design methodology of novel 3D SERS probes by quantifying formidable multiple coupling effects, i.e., deriving dimension, symmetry, Au roughness, and polarization dependences of intrinsic localized EMF enhancements based on 3D Au/SiO_2_ periodic hybrid nanogrid models. The methodology can be applied for the accurate design of high performance SERS probes with different plasmonic and dielectric materials by maximizing the contributions of the optical standing wave effects of incident light, SPP interference effects, LSPR effects and their coupling effects to intrinsic EMF enhancements, which is well demonstrated by SERS experiments. For the optimized structure the contribution of extra LSPR effects excited by SPP interference waves to the maximum and average intrinsic EF enhancements are even larger than that without the interference of SPP waves. Furthermore, the contribution ratio is independent of Au roughness, which is very unusual to be beyond the conventional knowledge and recognization. The methodology can not only provide the general design principles for high performance 3D SERS probe structures with any plasmonic metal/dielectric hybrids but also the accurate sizes along the horizontal and vertical directions of structures as follows. 1) Calculate SPP wavelengths based on the parameters of metal and dielectric materials and incident light; 2) adopt structures with high symmetry to improve detection uniformity due to the polarization dependences of LSPR effects and SPP wave coupling excitation effects; 3) set the height of 3D structure to be 36% wavelength of incident light, which is adequate for the achievement of the maximum intrinsic EMF enhancement along the height direction due to the optical standing wave effect, regardless of probe materials and structures; 4) create periodic cavity structures with parallel sidewalls and design the horizontal dimensions with integer multiples of half of SPP wavelengths to guarantee the generation of SPP waves and maximize FP resonance‐like interference effects to excite extra LSPR further; 5) determine a particular polarization direction relative to structure to enhance maximum intrinsic EMF for the optimization of detection limit; 6) increase metal surface roughness and/or narrow nanogaps of plasmonic particles as possible in terms of physical and/or chemical processes to increase intrinsic EMF further, which is independent of five items above.

## 3 Conclusion

Hexagonal 3D Au/SiO_2_ periodic nanogrids _h188 (30°) designed and optimized using the methodology above can detect the limit concentration of 2.17 × 10^−11^ m for R6G with 405 times improvement compared to the unoptimized h100 with the same number density of hot spots, 3.4 × 10^8^ SERS EF and RSD 5.52% detection uniformity, and can successfully detect 5.0 × 10^−11^ m trace Hg ions in water. Undoubtedly, the achievement of superior detection limit and uniformity for the SERS probes with the relative simple periodic nanostructures and low number density of hot spots is attributed to the optimized design using the proposed methodology. This study not only provides novel 3D Au/SiO_2_ periodic nanogrids as SERS probes with high performance, but also addresses the formidable issues of quantifying multiple coupling effects involved in SERS. The generalized methodology proposed here enables the accurate design of 3D SERS probe structures with high performance by combining state‐of‐the‐art top–down nanofabrication (even far cheaper nanoimprinting or 3D printing) and bottom–up technologies.

## 4 Experimental Section

*Design and Fabrication of Nanogrids*: 3D Au/SiO_2_ periodic nanogrids with various geometries and dimensions were designed and fabricated. Hydrogen silsesquioxane HSQ (XR‐1541‐006, Dow Corning, USA) was first spin‐coated on silicon (100) substrates with the thicknesses of 34, 58, 79, 108, 134, 163, and 198 nm for height control of nanogrids. Patterning was realized by using electron‐beam lithography (EBL, Vistec EBPG 5000 plus ES, Raith Company, Germany) with an accelerating voltage of 100 kV and a beam current of 2 nA, followed by the development. The typical width of SiO_2_ sidewalls for all nanogrids was controlled to be around 9 nm. Cr adhesion layers (3 nm thick) and Au films with the thicknesses of 1, 9, 18, 27, and 36 nm were deposited using an electron‐beam evaporator (OHMIKER‐50B, Cello‐Tech Company, Taiwan, China). As a reference sample, lift‐off was conducted by immersing the samples in a 1:5 buffered hydrofluoric acid (HF) solution (7:1 of 40% NH_4_F and 49% HF) at room temperature with ultrasonic agitation for 4 min. The fabricated structures were observed using a scanning electron microscope (NOVA NanoSEM 430, FEI Company, USA). Atomic force microscopy (AFM) images were acquired on a Veeco Dimension 3100 microscope (Veeco Digital Instruments, US). The schematic of sample fabrication is shown in Figure S1 of Supporting Information.

*SERS Measurements*: All the nanogrids with different symmetries and geometric dimensions were first immersed into Rhodamine 6G (R6G) aqueous solution with the concentrations ranging from 2.5 × 10^−11^ to 10^−5^ m for 12 h, and then dried naturally in air as SERS substrates. SERS measurements were performed on a Raman spectroscopy (Renishaw inVia, Renishaw company, UK) with a 50× objective (numerical aperture, NA = 0.75), a laser of 632.8 nm with a power of 0.5 mW, the *x*‐polarization and an integration time of 10 s. Likewise, SERS probes decorated with deionized water were used to measure noise with the same procedure.

*FDTD Calculations and Optical Measurements*: Finite‐difference time‐domain (FDTD) method was used to calculate the spatial distributions of the electromagnetic fields. For simplicity, the rough Au/SiO_2_ models with periodically arranged hemisphere‐ or semiellipsoid‐like Au nanoparticles on the sidewalls of SiO_2_ nanogrids were used to model the real Au/SiO_2_ nanogrids in which the sizes of Au particles were basically derived from SEM observations. Periodic boundary conditions for the *xz* and *yz* planes were applied to simulate an infinite array of periodic nanogrids or nanowalls. Perfectly matched layer (PML) boundary conditions were used in the *z*‐direction. The mesh size used in the simulation region was 2 nm for the calculations of SPP effects and 0.5 nm for the calculations of LSPR effects. The optical constants of exposed HSQ were determined using a spectroscopic ellipsometer (SE 850 DUV, Sentech Company, Germany). The infrared spectra were recorded on a Fourier transform infrared spectrometer (Nicolet iN10, Thermo Fisher Company, USA).

*Detection of Hg Ions*: The samples were first immersed into 4,4′‐Bipyridine (Bpy) absolute ethanol solution with a concentration of 10^−5^ m for 4 h, and then dried naturally in air as SERS probes for Hg ions detection. Hg ion solution (35 µL) with different concentrations of 5.0 × 10^−12^ (1 ppt), 5.0 × 10^−11^ (10 ppt), 5.0 × 10^−9^, 5.0 × 10^−7^, and 5.0 × 10^−5^ m was dropped onto the SERS probes, respectively, then kept for 10 min, and finally dried in air. Likewise, 35 µL of deionized water was prepared with the same procedure as SERS probes for the blank control group. The SERS measurements were performed using a 632.8 nm laser with a power of 1 mW and the *x*‐polarization on a Renishaw inVia Raman microscope equipped with a 20× objective (NA = 0.4) and an integration time of 10 s. For each sample, measurements on at least five different positions were taken.

## Conflict of Interest

The authors declare no conflict of interest.

## Supporting information

Supplementary
