# Supplementary material for: A Generalized Methodology of Designing 3D SERS Probes with Superior Detection Limit and Uniformity by Maximizing Multiple Coupling Effects
Source: Adv Sci (Weinh). 2019 Apr 4;6(11):1900177. doi: 10.1002/advs.201900177 (PMC6548962; doi:10.1002/advs.201900177)
Supplement: Supplementary file 1 — Supplementary [file ADVS-6-1900177-s001.pdf]

Copyright WILEY-VCH Verlag GmbH & Co. KGaA, 69469 Weinheim, Germany, 2019.

ADVANCED SCIENCE
Open Access

Supporting Information

for *Adv. Sci.,* DOI: 10.1002/advs.201900177

A Generalized Methodology of Designing 3D SERS Probes with Superior Detection Limit and Uniformity by Maximizing Multiple Coupling Effects

Yi Tian, Hanfu Wang, Lanqin Yan, Xianfeng Zhang, Attia Falak, Yanjun Guo, Peipei Chen, Fengliang Dong,* Lianfeng Sun,* and Weiguo Chu**

Copyright WILEY-VCH Verlag GmbH & Co. KGaA, 69469 Weinheim, Germany, 2019.

Supporting Information

A Generalized Methodology of Designing 3D SERS Probes with Superior Detection Limit and Uniformity by Maximizing Multiple Coupling Effects

Yi Tian, Hanfu Wang, Lanqin Yan, Xianfeng Zhang, Attia Falak, Yanjun Guo, Peipei Chen, Fengliang Dong,* Lianfeng Sun,* and Weiguo Chu**

Dr. Y. Tian, Dr. H. Wang, L. Yan, Dr. X. Zhang, A. Falak, Dr. Y. Guo, Dr. P. Chen, Dr. F. Dong, Prof. L. Sun, Prof. W. Chu
Nanofabrication Laboratory, CAS Key Laboratory for Nanosystems and Hierachical Fabrication, CAS Center for Excellence in Nanoscience, National Center for Nanoscience and Technology, Beijing 100190, P. R. China
E-mail: wgchu@nanoctr.cn; chenpp@nanoctr.cn; dongfl@nanoctr.cn; slf@nanoctr.cn
A. Falak, Dr. P. Chen, Dr. F. Dong, Prof. W. Chu
Center of Materials Science and Optoelectronics Engineering, University of Chinese Academy of Sciences,
Beijing 100049, P. R. China.
E-mail: wgchu@nanoctr.cn; chenpp@nanoctr.cn; dongfl@nanoctr.cn

Table S1. All the abbreviations with the corresponding indications.

Abbreviations	Indications
18, 27, 36 nm Au/198 nm SiO_2 _h200 (_s200, _t200)	18, 27 and 36 nm thick Au evaporated on 198 nm high SiO_2 hexagonal (square, triangular) nanogrids with a 200 nm grid length, respectively
h100, h200, h188, h188 (30°) and h274	Hexagonal nanogrids with a grid length of 100, 200, 188, 188 (with a polarization angle of 30°) and 274 nm, respectively, for all different Au thicknesses and SiO_2 heights
s174, s200, s325 and s475	Square nanogrids with a grid length of 174, 200, 325 and 475 nm, respectively, for all different Au thicknesses and SiO_2 heights
t200	Triangular nanogrids with a grid length of 200 nm, for all different Au thicknesses and SiO_2 heights
18, 27 and 36 nm Au/SiO_2 nanowalls _*D*200, *x*200 and *y*200	18, 27 and 36 nm thick Au evaporated on SiO_2 nanowalls with a wall center distance of 200 nm and different heights along arbitrary, *x* and *y*-direction, respectively
36 nm Au/198 nm SiO_2 nanogrids	Triangular, square and hexagonal 36 nm thick Au evaporated on 198 nm high SiO_2 nanogrids with different grid lengths

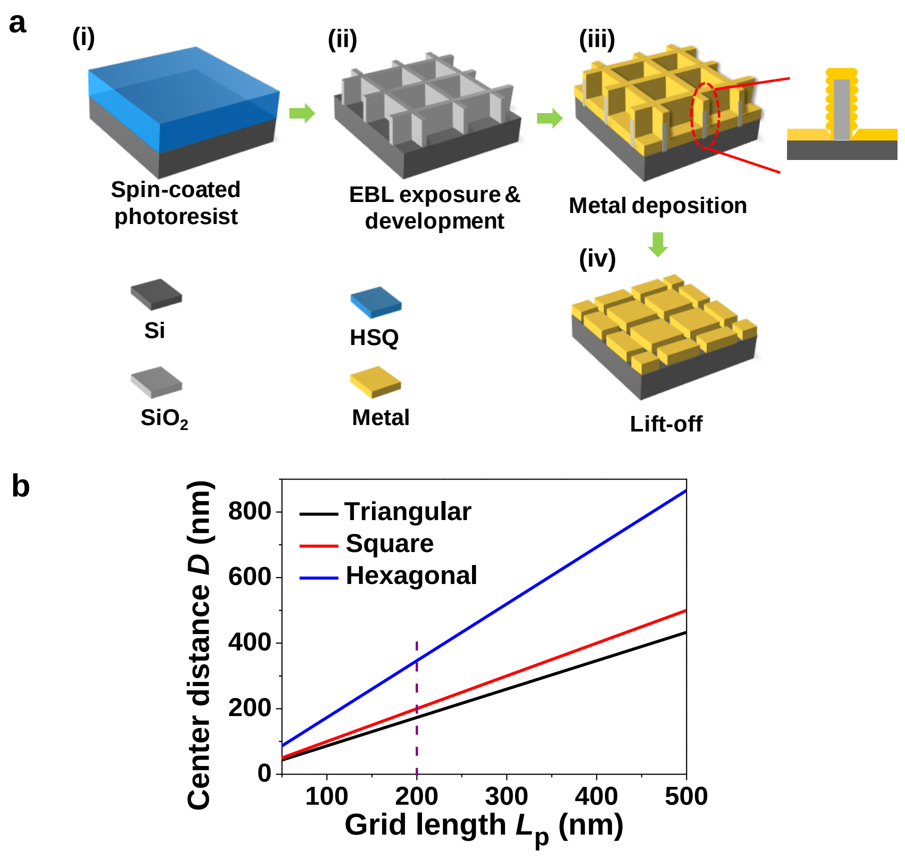

Figure S1. Fabrication schematics of 3D Au/SiO_2 periodic nanogrids, their corresponding nanostructures without sidewalls as the references and the relationships between grid length L_p and center distance D. a, Firstly, HSQ resist was spin-coated on Si substrates (i), followed by EBL and development (ii) according to designs. The development of exposed HSQ would convert the HSQ to SiO_2.[1,2] Afterwards, ultrathin Cr adhesion layers and Au films with various thicknesses were deposited using electron beam evaporation (iii), respectively. Finally, the corresponding nanostructures without sidewalls were prepared as the reference samples for comparison by using a lift-off process (iv). **b**, The relationships between grid length L_p and sidewall center distance D of triangular, square and hexagonal Au/SiO_2 nanogrids.

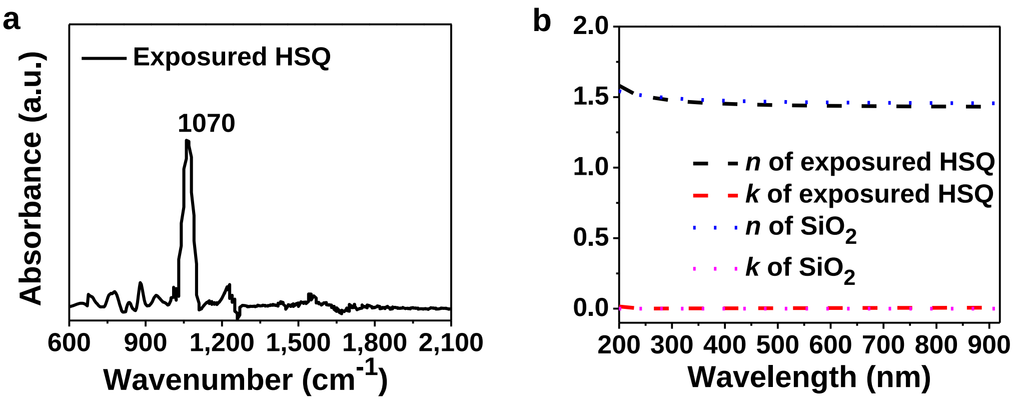

Figure S2. Fourier transform infrared spectrum of large-area exposed HSQ, and comparison of its optical constants and those of SiO_2. **a**, Fourier transform infrared (FTIR) spectrum of exposed HSQ, where 1070 cm^{-1} peak is a typical absorption peak of Si – O – Si network structures, characterized by the presence of SiO_2.[1,2] **b**, Comparison of optical constants of exposed HSQ derived from the measurements by spectroscopic ellipsometry and typical SiO_2.

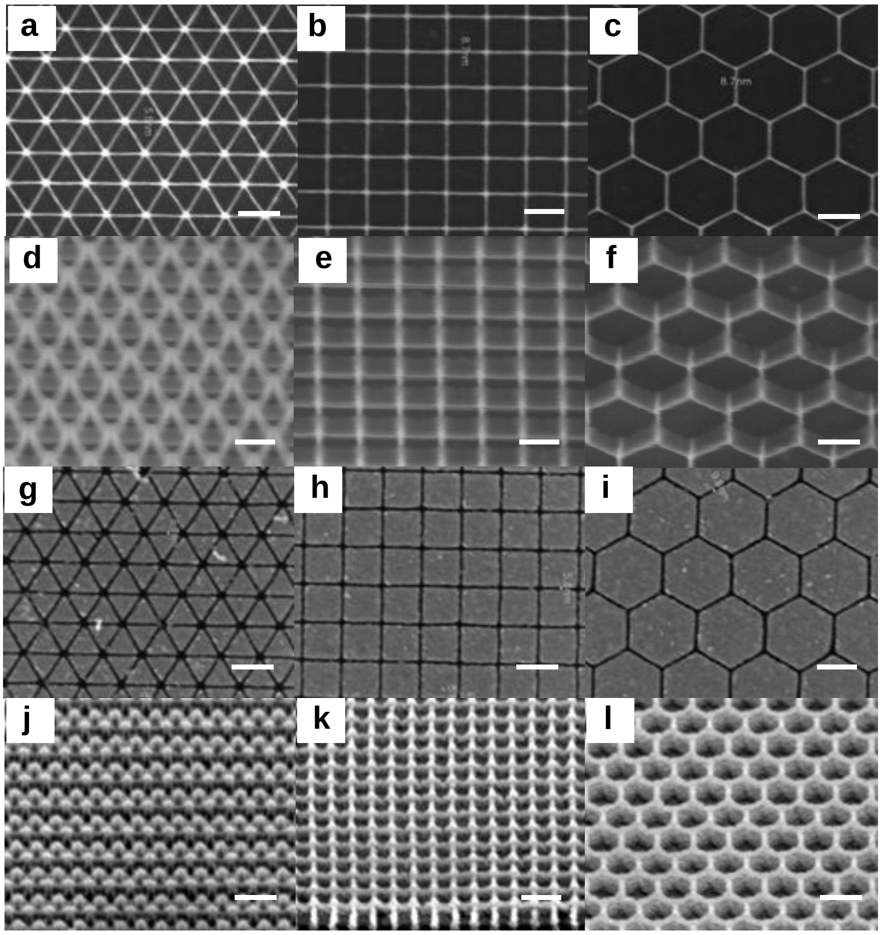

Figure S3. SEM images of triangular, square and hexagonal periodic SiO_2, Au/SiO_2 nanogrids and corresponding reference samples without sidewalls. a-c, SEM images of triangular, square and hexagonal SiO_2 nanogrids with 198 nm heights and 200 nm grid length. **d-f**, Corresponding tilt SEM images in **a-c**. **g-i**, SEM images of triangular, square and hexagonal 36 nm thick Au periodic nanostructures without sidewalls with 200 nm side length prepared by lift-off as the reference samples. **j-l**, Tilt SEM images of triangular, square and hexagonal 36 nm Au/198 nm SiO_2 nanogrids with 100 nm grid length. Scale bars: 200 nm.

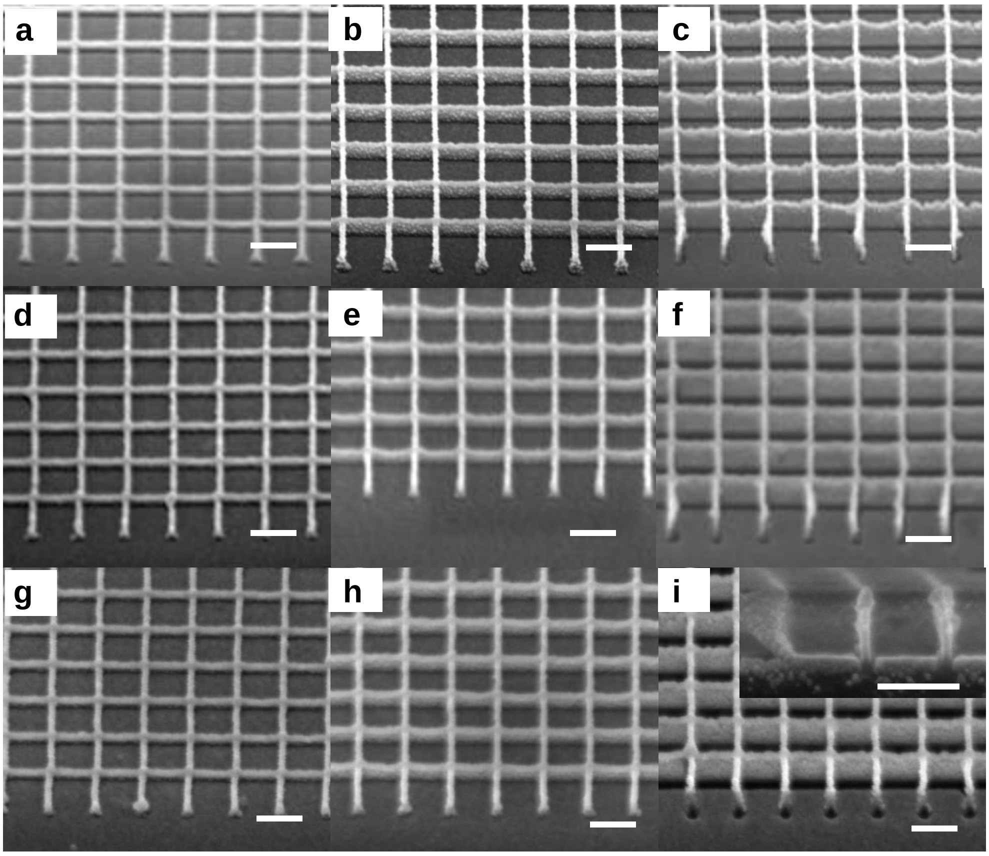

Figure S4. Tilt SEM images of square Au/SiO_2 nanogrids with 200 nm grid length, different Au thicknesses and SiO_2 heights. a-c, 18 nm Au/SiO_2 nanogrids with 58, 108 and 198 nm height, respectively. **d-f**, 27 nm Au/SiO_2 nanogrids with 58, 108 and 198 nm height, respectively. **g-i**, 36 nm Au/SiO_2 nanogrids with 58, 108 and 198 nm height, respectively. The inset in **i** shows SEM image of sidewalls of the 36 nm Au/198 nm SiO_2 nanogrids. Noting that with increased height and Au thickness the roughness tends to increase initially and decrease then. Scale bars: 200 nm.

S1. Geometry parameters of Au/SiO_2 nanogrids derived from statistical analysis of SEM images

The average width W_0 of SiO_2 sidewalls, the width W_1 and height H_1 of square Au/SiO_2 hybrid sidewalls with 200 nm grid length which are defined in Figure S5a were statistically extracted from SEM images (Figure S4). SiO_2 height H_0 dependences of these parameters and the cross sectional Au areas of sidewalls for 18, 27 and 36 nm thick Au without considering the roughness are constructed in Figure S5. The average width W_0 of bare SiO_2 sidewalls is found to be around 9 nm, and the width W_1 of hybrid sidewalls is between 15 and 25 nm for 18, 27 and 36 nm Au thickness, and tends to decrease with the increased H_0 for an Au thickness, as shown in Figure S5b. The average height H_1 of Au/SiO_2 hybrid nanogrid sidewalls and the height H_0 of bare SiO_2 nanogrid sidewalls are almost same (Figure S5c). Based on the geometrical relationship shown in Figure S5a, SiO_2 height H_0 dependences of the cross sectional Au area of sidewalls can be described as $S = H_1 (W_1 – W_0) \approx H_0 (W_1 − W_0)$, which are well fitted by the second order polynomial $S(H_0) = A + BH_0 + CH_0^2$ (Figure S5d) with the corresponding A, B and C values outlined in Table S2.

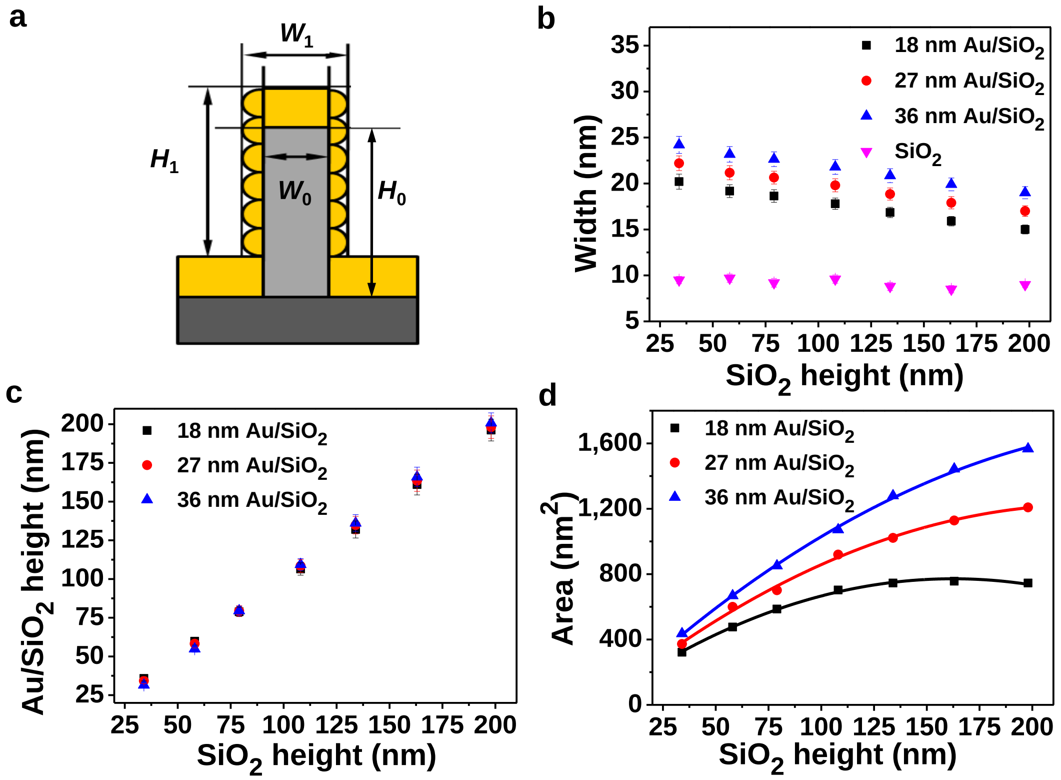

Figure S5. Geometry parameters of square 18, 27 and 36 nm Au/SiO_2 nanogrids with 200 nm grid length and various SiO_2 heights. a, Geometry parameters of nanogrids defined. **b**, Changes of average sidewall width W_1 of square Au/SiO_2 nanogrids and sidewall width W_0 of bare SiO_2 nanogrids with height. **c**, The relationships between the average height H_1 of Au/SiO_2 hybrid nanogrids and the height H_0 of bare SiO_2 nanogrids. **d**, The cross sectional Au areas of sidewalls versus the height of bare SiO_2 nanogrids from which the densities of Au nanoparticles can be derived for FDTD calculations.

Table S2. Coefficients of second order polynomial employed to fit the relationship between the cross section areas of Au sidewalls and height presented in Figure S5d.

Au thickness (nm)	*A*	*B*	*C*
**18**	58.73	8.77	-0.027
**27**	57.13	10.26	-0.0225
**36**	46.73	11.97	-0.0214

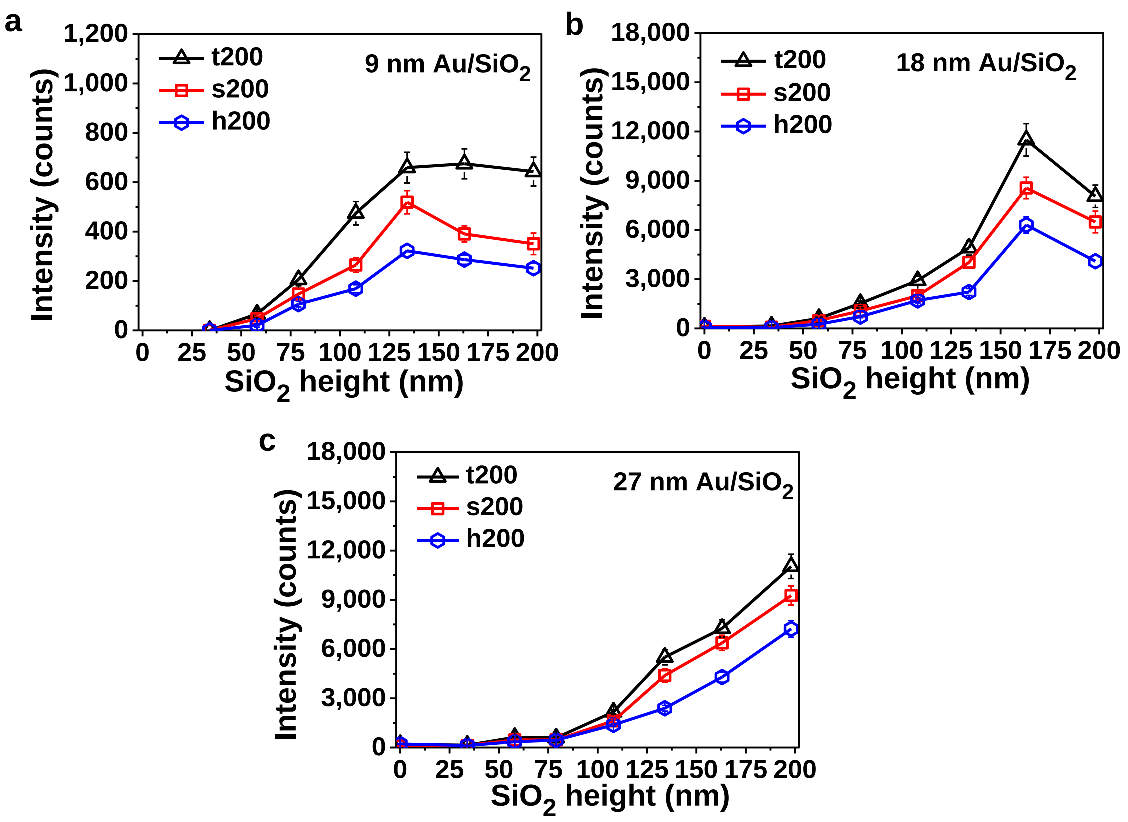

Figure S6. Changes of experimental Raman intensity of the peak positioned at 1360 cm^{-1} of the R6G - decorated triangular, square and hexagonal Au/SiO_2 nanogrids with 200 nm grid length versus SiO_2 nanogrid height. The heights are measured to be 34, 58, 79, 108, 134, 163 and 198 nm, respectively, which correspond to the nominal heights of 35, 60, 80, 110, 135, 165 and 200 nm, respectively. **a**, 9 nm thick Au. **b,** 18 nm thick Au. **c**, 27 nm thick Au. For both 27 and 36 nm thicknesses the intensity increases continuously with height whereas for 9 and 18 nm the intensity maxima are observed which shift to the larger height side for thicker Au.

S2. Calculations of SPP wavelength (λ_{SPP}) and SPP waves interference effects

It is well recognized that SPP can be excited by an incident light for a rough surface because the conditions of wavevector match for the incident light and SPP can be easily satisfied in the near field region owing to the random reflection in many directions.[3,4] As shown in Figure S7a, SPP1 for the model can be excited by a polarized light (near-field) with a component of its electric field (E_y) perpendicular to the surface of sidewalls (in the *xz*-plane) or (E_x) parallel to the propagation direction of SPP.[4] SPP2 can be excited by a polarized light with a component of its electric field (E_x) perpendicular to the nanowalls (along the *y*-direction), i.e. TM mode.

SPP on a metal film/dielectric interface propagate in the plane with the magnitude of wave vector [5]

$$k_{\mathrm{SPP}} = \frac{\omega}{c}\left(\frac{\varepsilon_{\mathrm{m}}\varepsilon_{\mathrm{d}}}{\varepsilon_{\mathrm{m}}+\varepsilon_{\mathrm{d}}}\right)^{1/2} \quad (1)$$

where ω and c are the frequency and speed of excitation light in free space, respectively, and ε_{m} and ε_{d} are the dielectric constants of metal and dielectric material (Au/SiO_2 and Au/air here), respectively. Then, the SPP wavelength, λ_{SPP}, can be obtained as

$$\lambda_{\mathrm{SPP}} = \lambda_{\mathrm{ex}}\left(\frac{\varepsilon_{\mathrm{m}}+\varepsilon_{\mathrm{d}}}{\varepsilon_{\mathrm{m}}\varepsilon_{\mathrm{d}}}\right)^{1/2} \quad (2)$$

where λ_{ex} is the wavelength of excitation source in free space. Accordingly, for $\lambda_{\mathrm{ex}} = 632.8$ nm, $\lambda_{\mathrm{SPP\ Au/air}}$ for Au/air interface is derived to be 603 nm with $\varepsilon_{\mathrm{m}} = -10.88$ [6] and $\varepsilon_{\mathrm{d}} = 1$, and $\lambda_{\mathrm{SPP\ Au/SiO_2}}$ for Au/SiO_2 interface is derived to be 389 nm with $\varepsilon_{\mathrm{m}} = -10.88$ and $\varepsilon_{\mathrm{d}} = 2.13$.

Both SPP1 and SPP2 waves can propagate not only in the positive *x* direction but also in the opposite direction (Figure S7a). The interference of two SPP waves with the opposite directions is not considered because there is no fixed phase difference between them. When the excited SPP wave propagates to encounter a sidewall, the partial reflection, transmission and scattering all would occur. Here, we reasonably consider only the reflected SPP wave

because we aim to study the interference of SPP wave confined in a single cavity. Once reflected, the SPP wave propagates again to encounter the sidewall to be partially reflected again. Thus, the multiple reflections of SPP wave take place in a single cavity to create a Fabry-Pérot (FP) resonance - like interference with an interference intensity of $I_{\text{SPP-interference}}$. The electric field intensity of SPP wave interference is

$$\begin{aligned} E_{\text{SPP-interference}}(x) &= E_{\text{SPP}}(x)+E_{\text{SPPr1}}(x)+E_{\text{SPPr2}}(x)+E_{\text{SPPr3}}(x)+E_{\text{SPPr4}}(x)+\cdots \\ &= A_{\text{SPP}}\left[\left(\sqrt{R}\right)^n \mathrm{e}^{\mathrm{i}\left((-1)^n k_{\text{SPP}}x-\omega t+nk_{\text{SPP}}L\right)}\Big|_{n=0,2,4\cdots}+\left(\sqrt{R}\right)^n \mathrm{e}^{\mathrm{i}\left((-1)^n k_{\text{SPP}}x-\omega t+\pi+(n+1)k_{\text{SPP}}L\right)}\Big|_{n=1,3,5\cdots}\right] \\ &= A_{\text{SPP}}\mathrm{e}^{\mathrm{i}(-\omega t)}\left[\left(\sqrt{R}\right)^n \mathrm{e}^{\mathrm{i}\left((-1)^n k_{\text{SPP}}x+nk_{\text{SPP}}L\right)}\Big|_{n=0,2,4\cdots}-\left(\sqrt{R}\right)^n \mathrm{e}^{\mathrm{i}\left((-1)^n k_{\text{SPP}}x+(n+1)k_{\text{SPP}}L\right)}\Big|_{n=1,3,5\cdots}\right] \end{aligned} \tag{3}$$

So, the interference intensity of SPP wave is

$$\begin{aligned} I_{\text{SPP-interference}}(x) &= \left|E_{\text{SPP-interference}}(x)\right|^2 = E_{\text{SPP-interference}}(x)E_{\text{SPP-interference}}{}^*(x) \\ &= A_{\text{SPP}}{}^2\left[\left(\sqrt{R}\right)^n \mathrm{e}^{\mathrm{i}\left((-1)^n k_{\text{SPP}}x+nk_{\text{SPP}}L\right)}\Big|_{n=0,2,4\cdots}-\left(\sqrt{R}\right)^n \mathrm{e}^{\mathrm{i}\left((-1)^n k_{\text{SPP}}x+(n+1)k_{\text{SPP}}L\right)}\Big|_{n=1,3,5\cdots}\right] \\ &\quad \left[\left(\sqrt{R}\right)^n \mathrm{e}^{\mathrm{i}\left((-1)^n k_{\text{SPP}}x+nk_{\text{SPP}}L\right)}\Big|_{n=0,2,4\cdots}-\left(\sqrt{R}\right)^n \mathrm{e}^{\mathrm{i}\left((-1)^n k_{\text{SPP}}x+(n+1)k_{\text{SPP}}L\right)}\Big|_{n=1,3,5\cdots}\right]^* \end{aligned} \tag{4}$$

where A_{SPP} is the amplitude of SPP wave, R is the reflectance of SPP wave reflected by the opposite sidewall of cavity and L is the cavity length.

One should bear in mind that the interference occurring at the hot spots would influence the intensity of SERS. Therefore, we consider the SPP1 wave interference effects at the surface (in the y = 0 plane) of nanowalls along the x-direction (x-nanowall) and the SPP2 wave interference effects at the surface of y-nanowalls (in the plane with x = 0) and x-nanowalls (in the plane with y = 0), which can further excite LSPR at the hot spots of the square nanogrid sidewalls besides the incident light (Figure S7a).

For the SPP1 wave interference effect in the plane with y = 0, we need to average the interference intensity of SPP1 wave from x = 0 to $x = L = L_s$ (Here, the cavity length equals the sidewall length L_s.) as follows

$$\bar{I}_{\text{SPP1-interference}} = \frac{1}{L_s}\int_0^{L_s} I_{\text{SPP1-interference}}(x) \tag{5}$$

For the SPP2 wave interference effect in the plane with $x = 0$, the interference intensity of SPP2 is $I_{\text{SPP2-interference}}(0)$. For the SPP2 wave interference effect in the plane with $y = 0$, we also need to average the interference intensity of SPP2 wave from $x = 0$ to $x = L = L_w$ (Here, the cavity length equals the sidewall spacing L_w.) as follows

$$\bar{I}_{\text{SPP2-interference}} = \frac{1}{L_w}\int_0^{L_w} I_{\text{SPP2-interference}}(x) \tag{6}$$

Upon calculating, $R = 0.29$ and 0.13 were taken from the FDTD calculations on the model with 7 nm thickness of Au on each side of an Au/SiO_2 sidewall for the SPP wavelengths of 603 and 389 nm, respectively. We calculated the penetration depth of about 27 nm in Au for Au/SiO_2 interface. Based on the exponential decay of intensity, the intensity of SPP1 wave at the Au/SiO_2 interface is figured out to be three fifths that of SPP1 at the Au/air interface with the SPP1 wave at the Au/SiO_2 interface travelling across 7 nm thick Au considered. Then, we calculated the ratio between the SPP1 wave interference intensity and the SPP1 wave intensity in the $y = 0$ plane against sidewall length, and that for SPP2 in the $x = 0$ and $y = 0$ planes against L_w/λ_{SPP2} by only considering the first seven polynomials of SPP wave interference items, as shown in Figure S7b - d. Clearly, the SPP wave interference is found to be similar to Fabry-Pérot resonance, and the intensity is a periodic function of the cavity length.

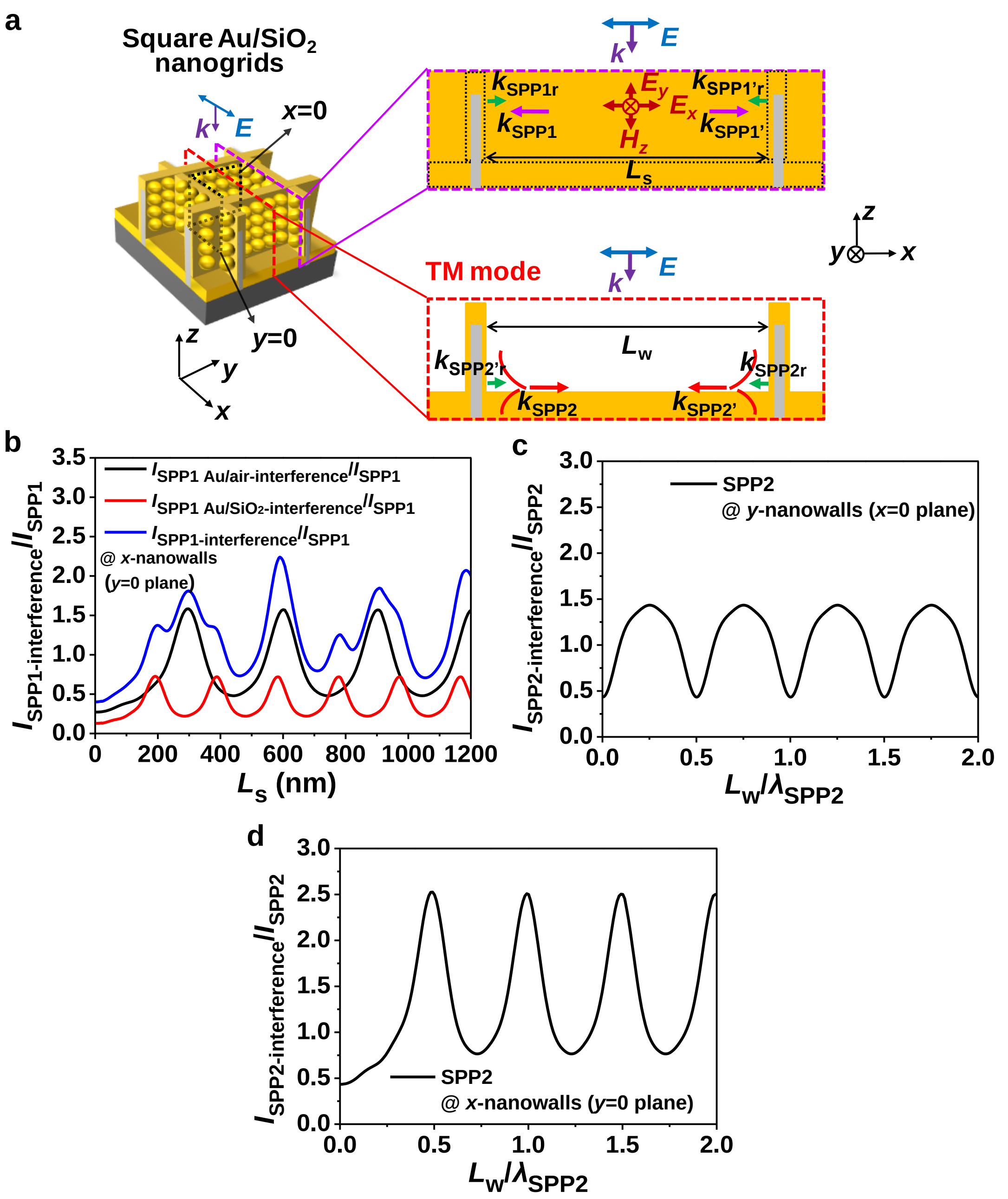

Figure S7. Mathematical analysis of SPP wave interference effects. a, Schematics of SPP1 and SPP2 waves interference effects. **b,** Theoretical ratio of the SPP1 wave interference intensity to the SPP1 wave intensity versus sidewall length L_s on the sidewall surface with y = 0. **c** and **d**, The ratios of the SPP2 wave interference intensity to the SPP2 wave intensity versus L_w/λ_{SPP2} on the sidewall surfaces with x = 0 and y = 0, respectively, in which L_w is the sidewall spacing.

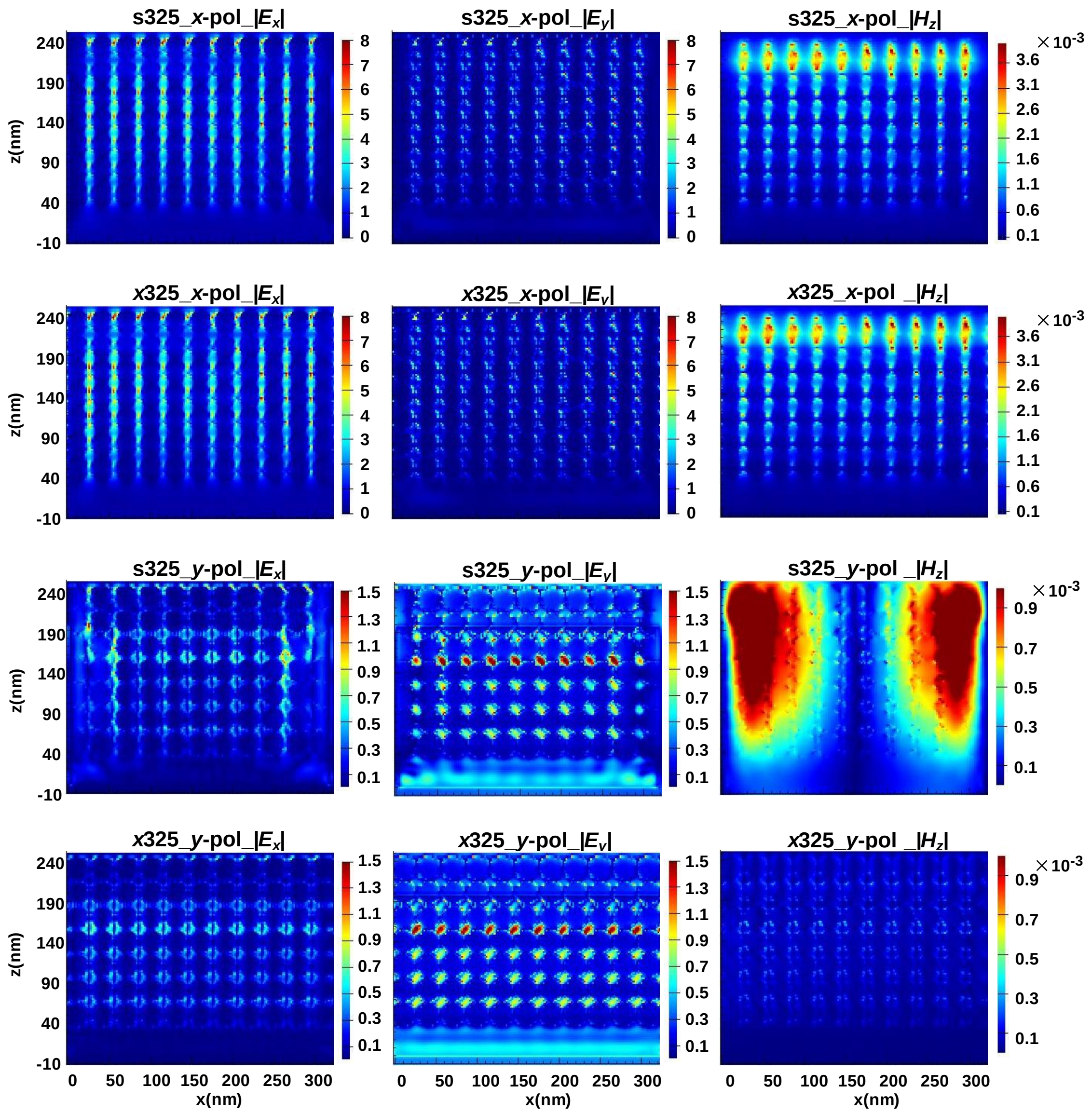

Figure S8. Calculated spatial distributions of the electromagnetic field components on the sidewall surfaces. Calculated spatial distributions of $|E_x|$, $|E_y|$ and $|H_z|$ on sidewall surfaces (parallel to the *xz* plane) of rough square 36 nm Au/198 nm SiO_2 nanogrids _s325 and nanowalls along *x*-direction _*x*325 with 325 nm sidewall center distance *D* (i.e. 302 nm sidewall length with 23 nm sidewall width) for *x* and *y*-polarized light (i.e. for the polarization angles α = 0 and π/2), respectively, by FDTD calculations. It is clear that SPP1 wave can be excited at the rough sidewalls and propagate along the *x*-direction for α = 0 and π/2. α is the angle between the nanowalls and the polarization direction of light.

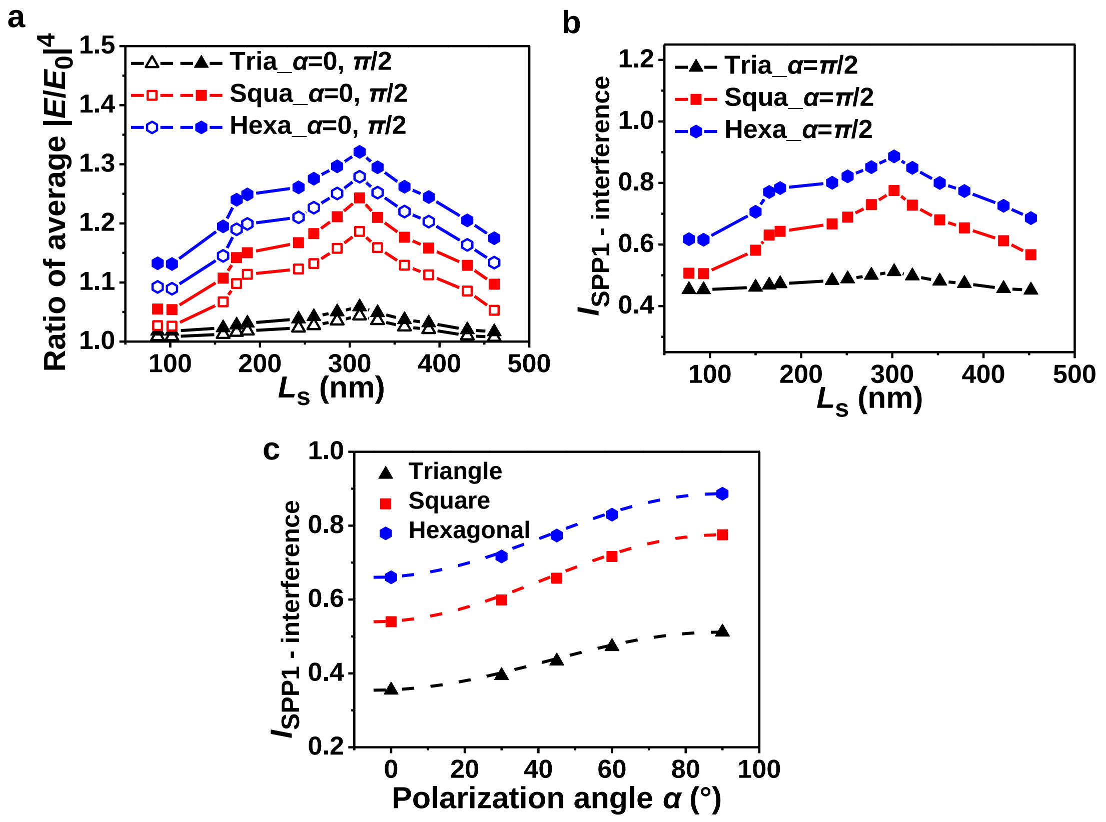

Figure S9. The relationships between SPP1 wave interference effects with sidewall length and polarization angle *α* calculated by FDTD calculations. a, The ratio of the average of the fourth power of electric field enhancements on one sidewall of the rough triangular, square and hexagonal 36 nm Au/198 nm SiO_2 nanogrids to those of nanowalls for $\alpha = 0$ and $\pi/2$ with the increased sidewall length L_s. **b**, Calculated interference intensities of SPP1 wave excited at the rough sidewalls of triangular, square and hexagonal 36 nm Au/198 nm SiO_2 nanogrids with the polarization angles of $\alpha = \pi/2$ against the increased sidewall length L_s. **c**, Calculated interference intensities of SPP1 wave taking place on a nanowall of triangular, square and hexagonal 36 nm Au/198 nm SiO_2 nanogrids with 302 nm sidewall length against polarization angle α, which can be well fitted with $(I_{1,\pi/2} - I_{1,0}) \sin^2\alpha + I_{1,0}$ ($I_{1,\pi/2}$ and $I_{1,0}$ is interference intensity of SPP1 wave for $\alpha = \pi/2$ and 0, respectively).

S3. Optical standing wave effect of the incident light

The incident light with *x*-polarization and the part reflected by the bottom gold surface of the hybrid nanogrids can form optical standing wave,[7] whose electric field intensity E_z shows the spatial distributions in the direction of height *z* as follows

$$\begin{aligned} E_z(z) &= E_0(z) + E_{0\mathrm{r}}(z) \\ &= E_0 \mathrm{e}^{\mathrm{i}(-kz-\omega t)} + \sqrt{R_z} E_0 \mathrm{e}^{\mathrm{i}(kz+\pi-\omega t)} \\ &= \mathrm{e}^{\mathrm{i}(-\omega t)} \left[(1-\sqrt{R_z}) E_0 \cos(kz) - \mathrm{i}(1+\sqrt{R_z}) E_0 \sin(kz) \right] \end{aligned} \tag{7}$$

The light intensity in height is

$$I_z(z) = \left| E_z(z) \right|^2 = (1+R_z) E_0^{\ 2} - 2\sqrt{R_z} E_0^{\ 2} \cos(2kz) \tag{8}$$

Thus, the normalized I_z is

$$\overline{I_z} = \frac{1}{z-z_0} \int_{z_0}^{z} I_z(z) = (1+\sqrt{R_z})^2 E_0^{\ 2} - \frac{\sqrt{R_z} E_0^{\ 2}}{kz} \sin(2kz) \tag{9}$$

where R_z, the reflectance of the incident light with *x*-polarization for square 36 nm Au/SiO_2 nanogrids with 200 nm grid length and a smooth 7 nm thick Au film on both sides of a sidewall, can be derived to be 0.47 from FDTD calculations. z_0, the coordinate position of the reflection plane, equals the thickness of Au film by defining the upper surface of silicon as the plane with z=0. Thus, we got the change of the normalized intensity of the optical standing wave with the ratio of height to the incident light wavelength, $(z-z_0)/\lambda$, which is shown in Figure S10.

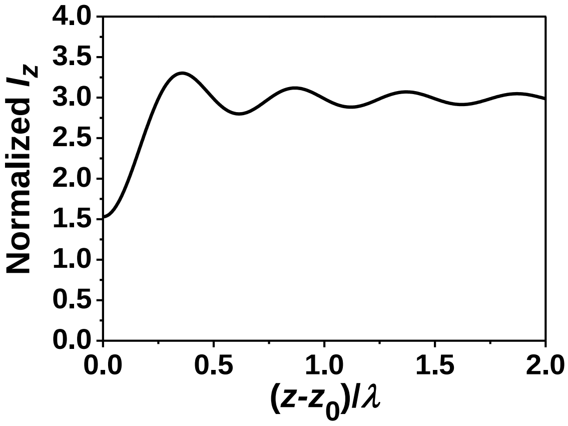

Figure S10. Change of normalized theoretical intensity of the optical standing wave formed by the interference between the incident light with *x*-polarization and the reflected part against the ratio of height to the incident light wavelength, $(z\text{-}z_0)/\lambda$. The oscillation behavior can be seen with the maximum interference intensity observed around 0.36 (i .e. $z\text{-}z_0$ = 228 nm). With the $(z\text{-}z_0)/\lambda$ larger than 0.36, the oscillation tends to be weakened evidently.

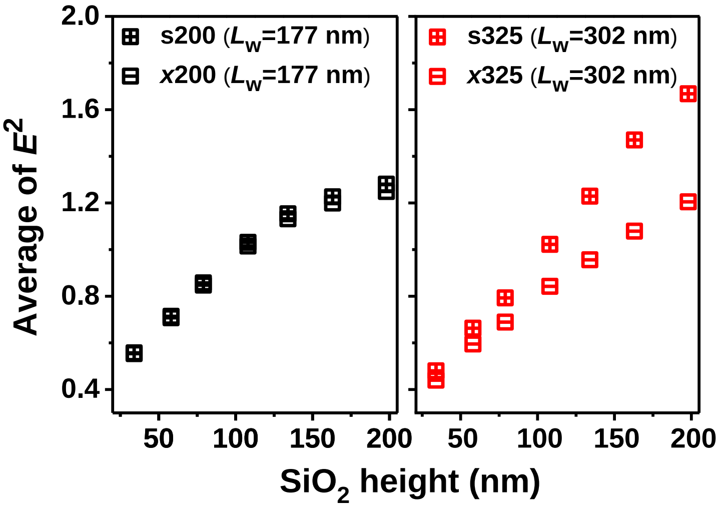

Figure S11. Calculated averages of the squared EF intensities at surfaces (parallel to the *xz* plane) of the smooth square 36 nm Au/SiO_2 nanogrids and *x*-nanowalls, with D = 200 nm and 325 nm (i.e. L_w = 177 nm and 302 nm, respectively) against SiO_2 height, respectively, which reveals the standing wave effect of incident light. Based on the average squared EF intensities of square nanogrids and *x*-nanowalls models, we calculated the changes of SPP2 interference intensity with SiO_2 height for s200 (L_w = 177 nm) and s325 (L_w = 302 nm), as shown in Figure 2f**.**

Table S3. The number *n* and center spacing *d* of, and the gap *g* between neighboring hemispheres / semiellipsoids Au nanoparticles for different SiO_2 heights and Au thicknesses derived from SEM and AFM observations.

**Au thickness (nm)**	**Parameters**	**SiO_2 height (nm)**						
		**34**	**58**	**79**	**108**	**134**	**163**	**198**
**18**	***n***	5	8	10	12	14	17	19
	***d* (nm)**	6.3	7.1	7.9	9.0	9.5	9.9	10.4
	***g* (nm)**	-3.7	-2.9	-2.1	-1.0	-0.5	0.1	0.4
**27**	***n***	4	6	8	10	12	14	17
	***d* (nm)**	7.7	8.7	9.7	10.8	11.2	11.6	11.8
	***g* (nm)**	-4.3	-3.3	-2.3	-1.2	-0.8	-0.4	-0.2
**36**	***n***	4	6	7	9	11	13	15
	***d* (nm)**	8.6	9.4	10.3	11.4	11.9	12.6	12.8
	***g* (nm)**	-4.4	-3.6	-2.7	-1.6	-1.1	-0.4	-0.2

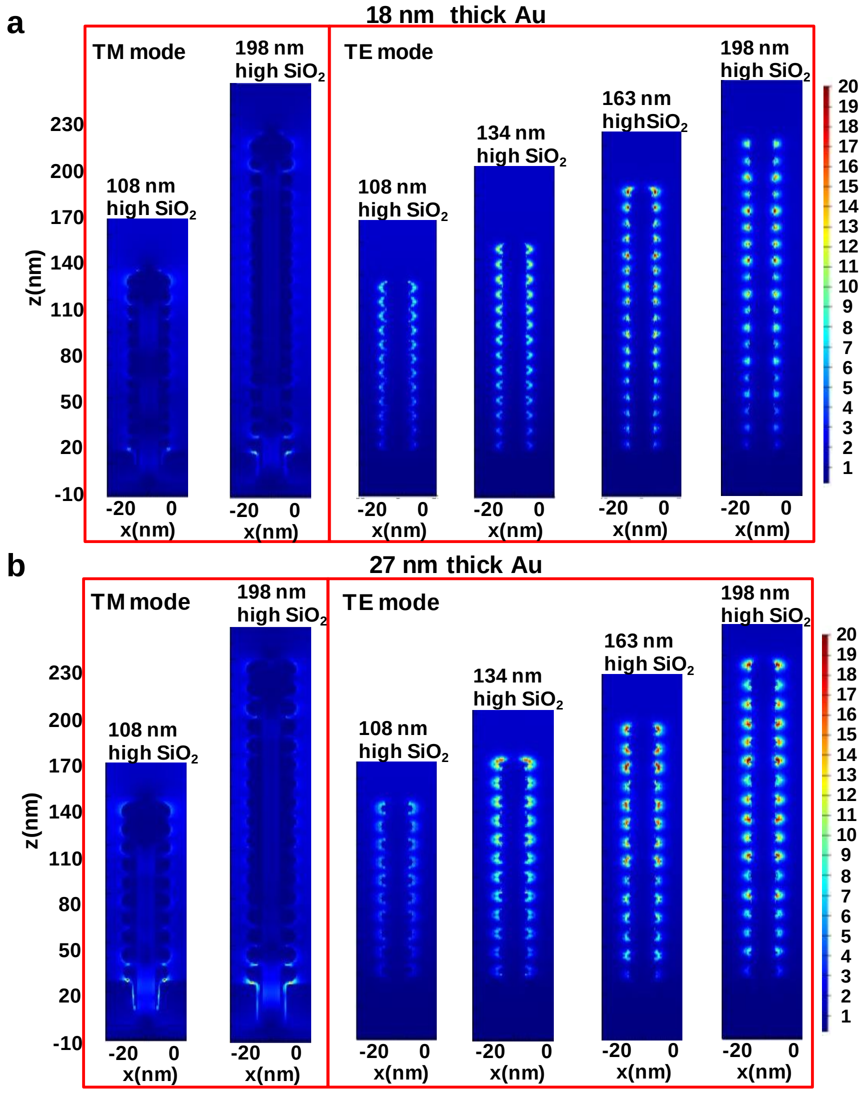

Figure S12. Calculated spatial distributions of the electric field intensities on the cross sections parallel to the *xz* plane for 18 and 27 nm Au/SiO_2 *y*-nanowalls with 200 nm center distance *D* and different heights for TM and TE modes. a, For 18 nm thick Au the maximum localized electric field intensity considering SPP1 coupling with LSPR2 is observed at the SiO_2 height of 163 nm for TE mode**.** Here, hemisphere-like Au nanoparticles with an average radius of 5 nm are taken for FDTD calculations. **b,** For 27 nm thick Au the change of the localized electric field with the increased height is similar to that for 36 nm thick Au, but with the lower values. Semiellipsoid-like Au nanoparticles with the average lengths of semi-principal axes, $a = b = 6$, and $c = 6.5$ nm, are employed for FDTD calculations.

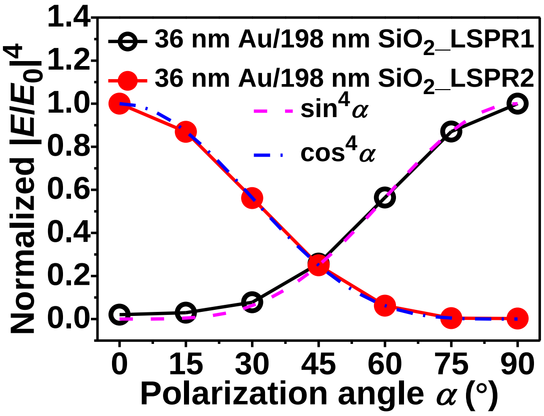

Figure S13. The normalized $|E/E_0|^4$ for LSPR1 and LSPR2 of rough 36 nm Au/198 nm SiO_2 nanowalls with 151 nm sidewall spacing L_w as a function of polarization angle α (solid line). The relationships can be described as $\sin^4\alpha$ (dashed line) and $\cos^4\alpha$ (dashed dot line), respectively.

S4. Coupling coefficients of different nanogrids

To get the averaged $|E/E_0|^4$ for triangular, square and hexagonal nanogrids (Figure S14), the values of the average $|E/E_0|^4$ of LSPR1 and LSPR2 for TM and TE mode, respectively (Figure 3d) multiplied by their respective coupling coefficients are added together to be shown in Figure 4a-4c. These coupling coefficients were closely related to the symmetry and dimension of nanogrids and the polarization of incident light. The results by theoretical analyses are shown in Table S4 and Figure S15.

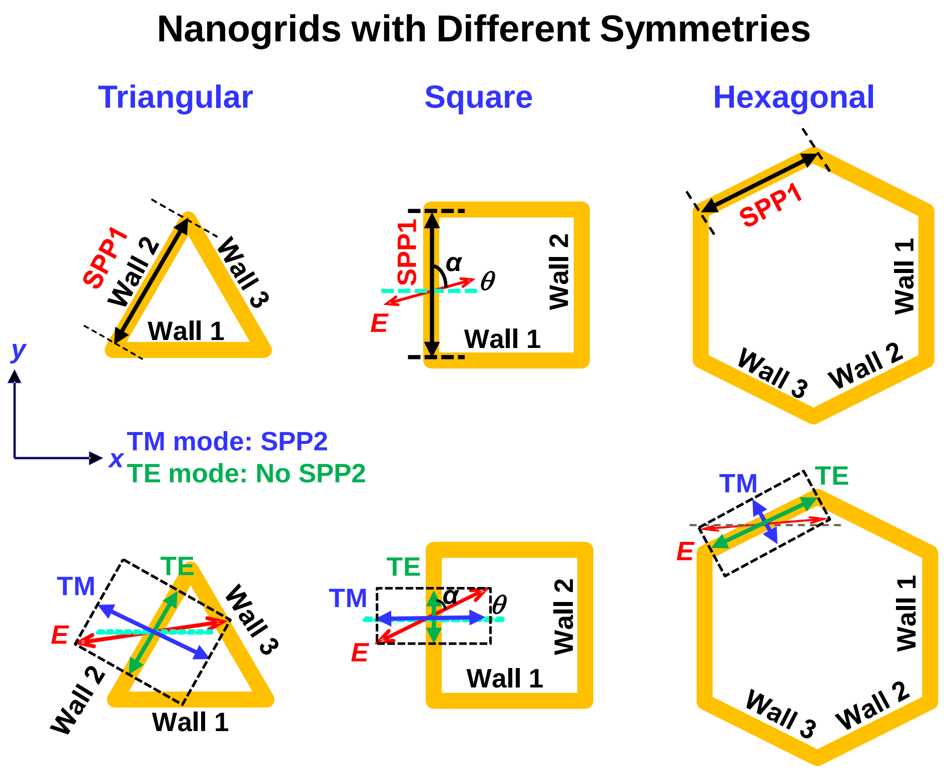

Figure S14. Models of triangular, square and hexagonal nanogrids for calculations of the averaged $|E/E_0|^4$ with SPP wave coupling excitation effects considered. Here, polarization angle θ and α is the angle between the polarization direction of light and x-direction, and between the nanowalls and the polarization direction of light, respectively.

Table S4. Polarization angle θ dependences of the average $|E/E_0|^4$ for triangular, square and hexagonal nanogrids.

**Triangular** [a)]		**Wall 1** [a),b)]	**Wall 2** [a),b)]	**Wall 3** [a),b)]
**Wall 1**<br>$\alpha = -\theta$	**TM mode**	$E_{LSPR1}{}^4\sin^4(-\theta)\,(I_{11}+1)$		
	**TE mode**	$E_{LSPR2}{}^4\cos^4(-\theta)\,(I_{11}+1)$		
**Wall 2**<br>$\alpha = \pi/3-\theta$	**TM mode**		$E_{LSPR1}{}^4\sin^4(\pi/3-\theta)\,(I_{12}+1)$	
	**TE mode**		$E_{LSPR2}{}^4\cos^4(\pi/3-\theta)\,(I_{12}+1)$	
**Wall 3**<br>$\alpha = -\pi/3-\theta$	**TM mode**			$E_{LSPR1}{}^4\sin^4(-\pi/3-\theta)(I_{13}+1)$
	**TE mode**			$E_{LSPR2}{}^4\cos^4(-\pi/3-\theta)(I_{13}+1)$
**Square**		**Wall 1**	**Wall 2**	
**Wall 1**<br>$\alpha = -\theta$	**TM mode**	$E_{LSPR1}{}^4\sin^4(-\theta)\,(I_{11}+1)$	$E_{LSPR1}{}^4\sin^4(\pi/2-\theta)$<br>$\bullet(I_{12}+1)I_2\cos^2(\pi/2-\theta)$<br>$E_{LSPR2}{}^4\cos^4(\pi/2-\theta)$<br>$\bullet(I_{12}+1)I_2\cos^2(\pi/2-\theta)$	
	**TE mode**	$E_{LSPR2}{}^4\cos^4(-\theta)\,(I_{11}+1)$		
**Wall 2**<br>$\alpha = \pi/2-\theta$	**TM mode**	$E_{LSPR1}{}^4\sin^4(-\theta)$<br>$\bullet\,(I_{11}+1)I_2\cos^2(\theta)$<br>$E_{LSPR2}{}^4\cos^4(-\theta)$<br>$\bullet\,(I_{11}+1)I_2\cos^2(\theta)$	$E_{LSPR1}{}^4\sin^4(\pi/2-\theta)\,(I_{12}+1)$	
	**TE mode**		$E_{LSPR2}{}^4\cos^4(\pi/2-\theta)\,(I_{12}+1)$	
**Hexagonal**		**Wall 1**	**Wall 2**	**Wall 3**
**Wall 1**<br>$\alpha = \pi/2-\theta$	**TM mode**	$E_{LSPR1}{}^4\sin^4(\pi/2-\theta)\,(I_{11}+1)$	$E_{LSPR1}{}^4\sin^4(\pi/6-\theta)$<br>$\bullet(I_{12}+1)I_2\cos^2(\theta)$<br>$E_{LSPR2}{}^4\cos^4(\pi/6-\theta)$<br>$\bullet\,(I_{12}+1)I_2\cos^2(\theta)$	$E_{LSPR1}{}^4\sin^4(-\pi/6-\theta)$<br>$\bullet\,(I_{13}+1)I_2\cos^2(\theta)$<br>$E_{LSPR2}{}^4\cos^4(-\pi/6-\theta)$<br>$\bullet\,(I_{13}+1)I_2\cos^2(\theta)$
	**TE mode**	$E_{LSPR2}{}^4\cos^4(\pi/2-\theta)\,(I_{11}+1)$		
**Wall 2**<br>$\alpha = \pi/6-\theta$	**TM mode**	$E_{LSPR1}{}^4\sin^4(\pi/2-\theta)$<br>$\bullet(I_{11}+1)I_2\cos^2(-\pi/3-\theta)$<br>$E_{LSPR2}{}^4\cos^4(\pi/2-\theta)$<br>$\bullet(I_{11}+1)I_2\cos^2(-\pi/3-\theta)$	$E_{LSPR1}{}^4\sin^4(\pi/6-\theta)\,(I_{12}+1)$	$E_{LSPR1}{}^4\sin^4(-\pi/6-\theta)$<br>$\bullet\,(I_{13}+1)I_2\cos^2(-\pi/3-\theta)$<br>$E_{LSPR2}{}^4\cos^4(-\pi/6-\theta)$<br>$\bullet(I_{13}+1)I_2\cos^2(-\pi/3-\theta)$
	**TE mode**		$E_{LSPR2}{}^4\cos^4(\pi/6-\theta)\,(I_{12}+1)$	
**Wall 3**<br>$\alpha = -\pi/6-\theta$	**TM mode**	$E_{LSPR1}{}^4\sin^4(\pi/2-\theta)$<br>$\bullet\,(I_{11}+1)I_2\cos^2(\pi/3-\theta)$<br>$E_{LSPR2}{}^4\cos^4(\pi/2-\theta)$<br>$\bullet(I_{11}+1)I_2\cos^2(\pi/3-\theta)$	$E_{LSPR1}{}^4\sin^4(\pi/6-\theta)$<br>$\bullet\,(I_{12}+1)I_2\cos^2(\pi/3-\theta)$<br>$E_{LSPR2}{}^4\cos^4(\pi/6-\theta)$<br>$\bullet\,(I_{12}+1)I_2\cos^2(\pi/3-\theta)$	$E_{LSPR1}{}^4\sin^4(-\pi/6-\theta)(I_{13}+1)$
	**TE mode**			$E_{LSPR2}{}^4\cos^4(-\pi/6-\theta)(I_{13}+1)$

[a)] Polarization angles θ and α is the angle between the polarization direction of light and x-direction, and between the nanowalls and the polarization direction of light, respectively (see Figure S14);
[b)] I_{1i} (i = 1, 2 and 3) are the interference intensities of SPP1 wave for walls1, 2 and 3, respectively (see Figure 2b, S9b, S9c and S14) and I_2 is the interference intensity of SPP2 wave for TM mode shown in Figure 2e.

Based on Table S4 and the light with the polarization angle of θ, the coupling coefficients of LSPR1 and LSPR2 of triangular, square and hexagonal nanogrids are derived to be

$$
\begin{aligned}
C_{\text{LSPR1-tria}\theta} &= [\sin^4\theta(\Delta I_1 \sin^2(0-\theta) + I_{1,0}+1) \\
&+ \sin^4(\frac{\pi}{3}-\theta)(\Delta I_1 \sin^2(\frac{\pi}{3}-\theta) + I_{1,0}+1) + \sin^4(\frac{-\pi}{3}-\theta)(\Delta I_1 \sin^2(\frac{-\pi}{3}-\theta) + I_{1,0}+1)]/3 \\
C_{\text{LSPR1-squa}\theta} &= 2[\sin^4\theta(1+I_2\cos^2\theta)(\Delta I_1 \sin^2(0-\theta) + I_{1,0}+1) \\
&+ \sin^4(\frac{\pi}{2}-\theta)(1+I_2\cos^2(\frac{\pi}{2}-\theta))(\Delta I_1 \sin^2(\frac{\pi}{2}-\theta) + I_{1,0}+1)]/4 \\
C_{\text{LSPR1-hexa}\theta} &= 2[\sin^4(\frac{\pi}{2}-\theta)[1+I_2(\cos^2(\frac{-\pi}{3}-\theta)+\cos^2(\frac{\pi}{3}-\theta))](\Delta I_1 \sin^2(\frac{\pi}{2}-\theta) + \\
&I_{1,0}+1) + \sin^4(\frac{\pi}{6}-\theta)[1+I_2(\cos^2\theta+\cos^2(\frac{\pi}{3}-\theta))](\Delta I_1 \sin^2(\frac{\pi}{6}-\theta) + I_{1,0}+1) \\
&+ \sin^4(\frac{-\pi}{6}-\theta)[1+I_2(\cos^2\theta+\cos^2(\frac{-\pi}{3}-\theta))](\Delta I_1 \sin^2(\frac{-\pi}{6}-\theta) + I_{1,0}+1)]/6
\end{aligned}
\quad (10)
$$

and

$$
\begin{aligned}
C_{\text{LSPR2-tria}\theta} &= [\cos^4\theta(\Delta I_1 \sin^2(0-\theta) + I_{1,0}+1) \\
&+ \cos^4(\frac{\pi}{3}-\theta)(\Delta I_1 \sin^2(\frac{\pi}{3}-\theta) + I_{1,0}+1) + \cos^4(\frac{-\pi}{3}-\theta)(\Delta I_1 \sin^2(\frac{-\pi}{3}-\theta) + I_{1,0}+1)]/3 \\
C_{\text{LSPR2-squa}\theta} &= 2[\cos^4\theta(1+I_2\cos^2\theta)(\Delta I_1 \sin^2(0-\theta) + I_{1,0}+1) \\
&+ \cos^4(\frac{\pi}{2}-\theta)(1+I_2\cos^2(\frac{\pi}{2}-\theta))(\Delta I_1 \sin^2(\frac{\pi}{2}-\theta) + I_{1,0}+1)]/4 \\
C_{\text{LSPR2-hexa}\theta} &= 2[\cos^4(\frac{\pi}{2}-\theta)[1+I_2(\cos^2(\frac{-\pi}{3}-\theta)+\cos^2(\frac{\pi}{3}-\theta))](\Delta I_1 \sin^2(\frac{\pi}{2}-\theta) + \\
&I_{1,0}+1) + \cos^4(\frac{\pi}{6}-\theta)[1+I_2(\cos^2\theta+\cos^2(\frac{\pi}{3}-\theta))](\Delta I_1 \sin^2(\frac{\pi}{6}-\theta) + I_{1,0}+1) \\
&+ \cos^4(\frac{-\pi}{6}-\theta)[1+I_2(\cos^2\theta+\cos^2(\frac{-\pi}{3}-\theta))](\Delta I_1 \sin^2(\frac{-\pi}{6}-\theta) + I_{1,0}+1)]/6
\end{aligned}
\quad (11)
$$

Here, $\Delta I_1 = I_{1,\alpha=\pi/2} - I_{1,\alpha=0}$ and $I_{1,0} = I_{1,\alpha=0}$, in which $I_{1,\alpha=0}$ and $I_{1,\alpha=\pi/2}$ are the interference intensities of SPP1 wave for α = 0 and π/2, respectively (Figure 2b and Figure S9b), and I_2 is the interference intensity of SPP2 wave for TM mode (Figure 2e).

Then, we can get the averaged $|E/E_0|^4$ of these nanogrids using the following formula

$$
\overline{|E_\theta/E_0|^4} = C_{\text{LSPR1-pattern}\theta}\overline{|E_{\text{LSPR1},\alpha=\pi/2}/E_0|^4} + C_{\text{LSPR2-pattern}\theta}\overline{|E_{\text{LSPR2},\alpha=0}/E_0|^4} \quad (12)
$$

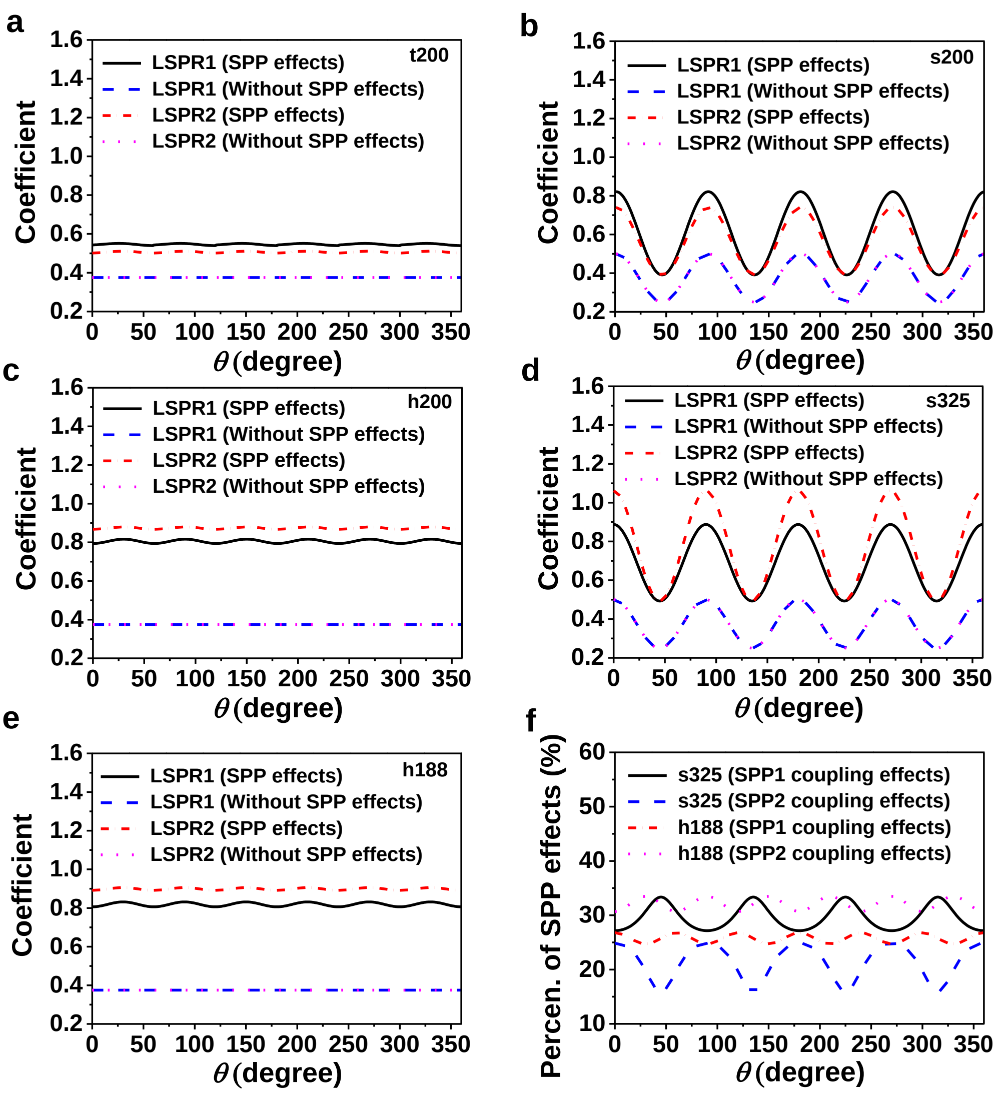

Figure S15. Polarization angle *θ* dependences of the coupling coefficients of different nanogrids and the corresponding contribution percentages of SPP wave coupling excitation effects. a-e, Polarization angle θ dependences of the coupling coefficients C_{LSPR1} and C_{LSPR2} of triangular, square and hexagonal nanogrids with and without the SPP wave coupling excitation effects considered. **f,** Percentages of intrinsic electric field enhancement derived from the SPP1 and SPP2 wave coupling excitation effects for square (s325) and hexagonal (h188) 36 nm Au/198 nm SiO_2 nanogrids versus polarization angle θ.

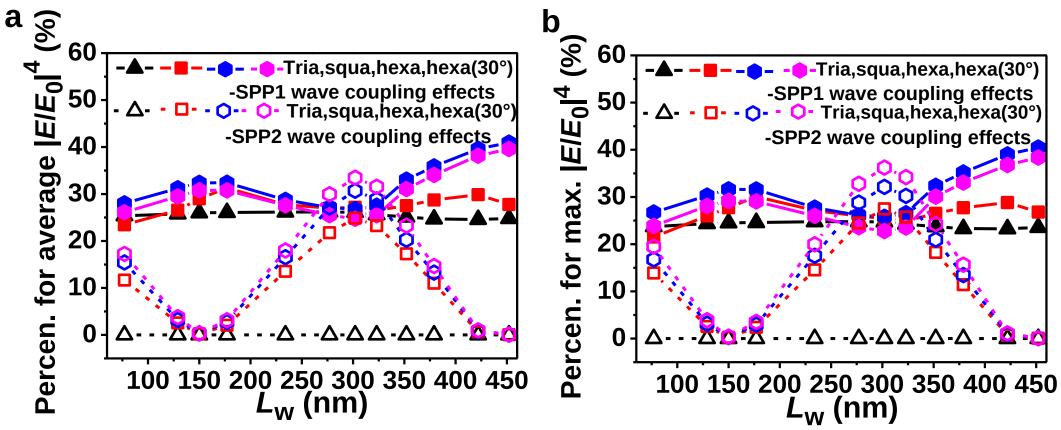

Figure S16. Sidewall spacing L_w dependences of the contributions of SPP1 and SPP2 wave coupling excitation effects to the average and maximum $|E/E_0|^4$ of different 36 nm Au/198 nm SiO_2 nanogrids, respectively. a, The contributions of SPP1 and SPP2 wave coupling excitation effects to the average $|E/E_0|^4$. **b,** The contributions of SPP1 and SPP2 wave coupling excitation effects to the maximum $|E/E_0|^4$.

Table S5. Comparison of theoretical results of multiple effects of triangular, square and hexagonal 3D Au/SiO_2 periodic nanogrids with the heights of 34 and 198 nm and various dimensions.

**Multiple different effects (Influencing factors)**		**Theoretical results**					
	Height of SiO_2 (nm)	**34**	**198**				
	Symmetry & grid length L_p (nm)	**s200**	**t200**	**s200**	**h200**	**s325*[a]**	**h188*[a]**
	Sidewall spacing L_w (nm)	**177**	**150**	**177**	**323**	**302**	**302**
**Excitation sources of nanogrids ($\|E/E_0\|^2$)**	**Optical standing wave (SiO_2 height)**	0.53		1.17			
	**SPP1 interference (Symmetry, sidewall length and polarization)**		0.325=[b]<br>0.477∥[b]	0.446=<br>0.647∥	0.557=<br>0.785∥	0.529=<br>0.768∥	0.556=<br>0.784∥
	**SPP2 interference (TM mode) (Sidewall spacing and SiO_2 height)**		0	0.024	0.347	0.38	0.38
		0.005		0.024			
**LSPR of nanowalls ($\|E/E_0\|^4$) (TM mode)**	**Maximum LSPR (α = 0) ($\times 10^6$) (Roughness and sidewall spacing)**	0.0055	3.40	3.96	5.06	5.02	5.02
	**Average LSPR (α = 0) ($\times 10^5$) (Roughness and sidewall spacing)**	0.0012	0.676	0.80	1.19	1.18	1.18
**Intrinsic electric field enhancement of nanogrids**	**Average $\|E/E_0\|^4$ ($\times 10^5$) (Symmetry, sidewall spacing and polarization)**		0.391	0.688	1.16<br>(θ=30°)	1.38	1.19<br>(θ=30°)
	**Average $\|E/E_0\|^4$ from SPP (%) (Symmetry, sidewall spacing and polarization)**		26.0	33.4	57.0<br>(θ=30°)	52.0	**58.2**<br>(θ=30°)
	**Maximum $\|E/E_0\|^4$ ($\times 10^7$) (Symmetry, sidewall spacing and polarization)**		0.451	0.586	1.20<br>(θ=30°)	1.07	1.22<br>(θ=30°)
	**Maximum $\|E/E_0\|^4$ from SPP (%) (Symmetry, sidewall spacing and polarization)**		24.5	32.5	57.8<br>(θ=30°)	52.9	**59.0**<br>(θ=30°)

[a] * represents the optimized structures;
[b] = and ∥ represent α = 0 and α = π/2, respectively.

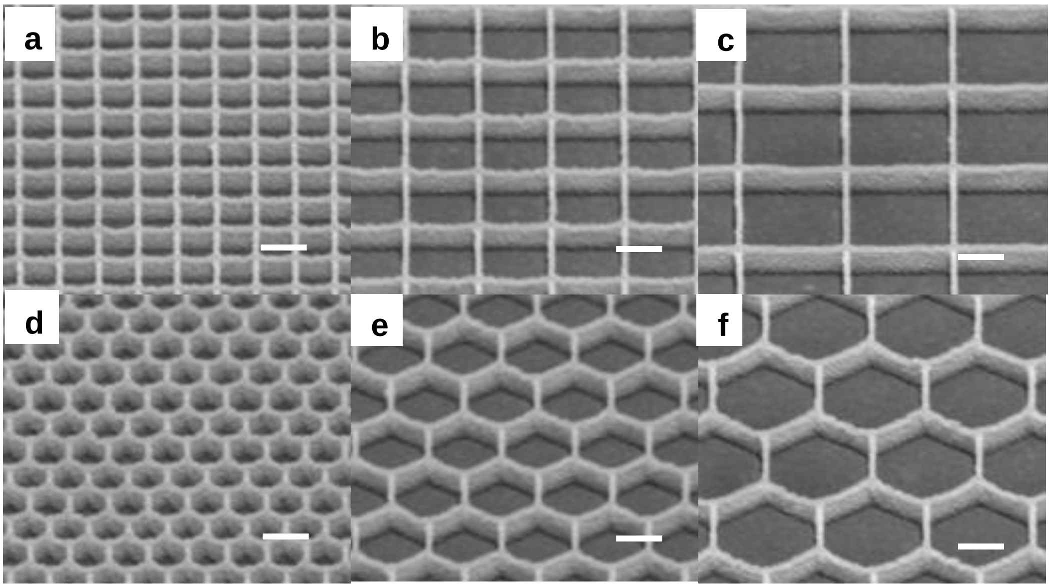

Figure S17. Tilt SEM images of square and hexagonal 36 nm Au/198 nm SiO_2 nanogrids with different sidewall spacings. a-c, Square nanogrids with 151, 302 and 452 nm sidewall spacing (i.e. s174, s325 and s475, respectively) with the sidewall width of 23 nm considered. **d-f,** Hexagonal nanogrids with the same sidewall spacings (i.e. h100, h188 and h274, respectively) with the sidewall width of 23 nm considered. Scale bars: 200 nm.

a

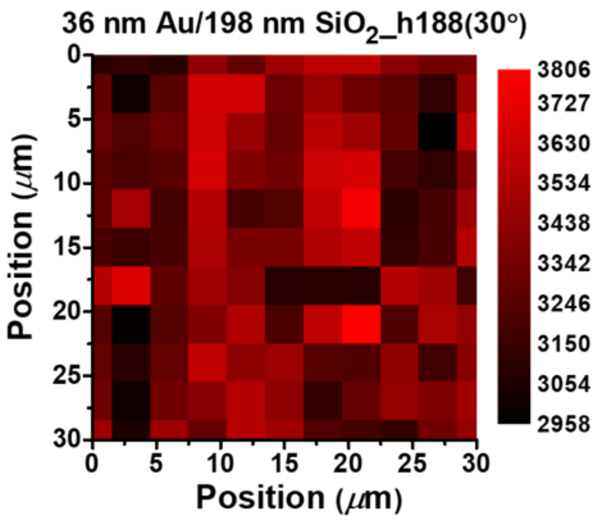

b

Intensity (counts)		Position (μm)										
		**0**	**3**	**6**	**9**	**12**	**15**	**18**	**21**	**24**	**27**	**30**
**Position (μm)**	**0**	3095	3137	3095	3432	3293	3479	3578	3586	3420	3331	3349
	**3**	3279	3008	3249	3648	3653	3322	3464	3324	3266	3124	3453
	**6**	3308	3228	3308	3635	3461	3297	3574	3493	3284	2959	3592
	**9**	3247	3207	3247	3661	3388	3326	3632	3664	3184	3119	3379
	**12**	3279	3505	3179	3556	3202	3232	3600	3773	3098	3196	3476
	**15**	3199	3153	3199	3532	3364	3352	3545	3588	3120	3202	3559
	**18**	3552	3688	3275	3489	3399	3087	3093	3087	3553	3485	3159
	**21**	3239	2975	3239	3382	3546	3209	3585	3804	3219	3526	3447
	**24**	3287	3086	3287	3599	3430	3480	3243	3223	3428	3171	3408
	**27**	3335	3007	3335	3411	3556	3433	3134	3290	3447	3384	3488
	**30**	3482	3047	3482	3294	3566	3494	3224	3189	3143	3341	3447

Figure S18. Raman intensity mapping of 10^{-5} M R6G decorated 36 nm Au/198 nm SiO_2 nanogrids _h188 (30°) as SERS probes. a, 121 data of intensities at 1360 cm^{-1} peak across an area of 30 μm × 30 μm were collected using a beam size of 1 μm at a step size of 3 μm upon SERS measurements with an integration time of 1 s. **b,** The intensities of corresponding 121 data. The nonuniformity is derived to be about 5.52%, which is quite good.

S5. Calculation of SERS enhancement factor

The SERS enhancement factor (SERS EF) can be estimated by the following equation:[8]

$$\mathrm{EF} = \frac{C_0 \times I_{\mathrm{SERS}}}{I_0 \times C_{\mathrm{SERS}}} \qquad (13)$$

where I_{SERS} and I_0 are the Raman scattering intensities of the peak at 1360 cm^{-1} for R6G for hexagonal 36 nm Au/198 nm SiO_2 nanogrids _h188 with the polarization angle of 30° and 36 nm thick Au film, respectively, and C_{SERS} and C_0 are the molar concentrations of R6G aqueous solution, 2.5×10^{-11} and 10^{-2} M used in this study, respectively, from which the SERS EF was derived to be 3.4×10^{8}. Therefore, the optimization of probes leads to a high SERS EF. The SERS EF calculated using the method in the manuscript better reflects the enhancement effect by the maximum electromagnetic field instead of the average enhancement effect due to the very low concentration molecules, 2.5×10^{-11} M, which are probably decorated at those hot spots with the maximum or stronger electromagnetic field enhancement.

Reference

[1] C. C. Yang, W. C. Chen, *J. Mater. Chem.* **2002**, *12*, 1138.
[2] T. Siegfried, Y. Ekinci, O. J. Martin, H. Sigg, *ACS Nano* **2013**, *7*, 2751.
[3] L. Salomon, G. Bassou, H. Aourag, J. P. Dufour, F. De Fornel, F. Carcenac, A. V. Zayats, *Phys. Rev. B* **2002**, *65*, 125409.
[4] A. V. Zayats, I. I. Smolyaninov, A. A. Maradudin, *Phys. Rep.* **2005**, *408*, 131.
[5] H. Raether, *Surface Plasmons on Smooth Surfaces*, Springer, Berlin, Heidelberg, Germany **1988**.
[6] F. Hao, P. Nordlander, *Chem. Phys. Lett.* **2007**, *446*, 115.
[7] H. J. Büchner, H. Stiebig, V. Mandryka, E. Bunte, G. Jäger, *Meas. Sci. Technol.* **2003**, *14*, 311.
[8] E. C. Le Ru, E. Blackie, M. Meyer, P. G. Etchegoin, *J. Phys. Chem. C* **2007**, *111*, 13794.
